# Structural and Genomic Insights Into Pyrazinamide Resistance in *Mycobacterium tuberculosis* Underlie Differences Between Ancient and Modern Lineages

**DOI:** 10.3389/fmolb.2021.619403

**Published:** 2021-07-23

**Authors:** Tanushree Tunstall, Jody Phelan, Charlotte Eccleston, Taane G. Clark, Nicholas Furnham

**Affiliations:** ^1^Department of Infection Biology, London School of Hygiene and Tropical Medicine, London, United Kingdom; ^2^Department of Infectious Disease Epidemiology, London School of Hygiene and Tropical Medicine, London, United Kingdom

**Keywords:** *Mycobacterium tuberculosis*, pncA, nsSNPs, non-synonymous Single Nucleotide Polymorphisms, biophysical effects, thermodynamic stability, mCSM, FoldX

## Abstract

Resistance to drugs used to treat tuberculosis disease (TB) continues to remain a public health burden, with missense point mutations in the underlying *Mycobacterium tuberculosis* bacteria described for nearly all anti-TB drugs. The post-genomics era along with advances in computational and structural biology provide opportunities to understand the interrelationships between the genetic basis and the structural consequences of *M. tuberculosis* mutations linked to drug resistance. Pyrazinamide (PZA) is a crucial first line antibiotic currently used in TB treatment regimens. The mutational promiscuity exhibited by the *pncA gene* (target for PZA) necessitates computational approaches to investigate the genetic and structural basis for PZA resistance development. We analysed 424 missense point mutations linked to PZA resistance derived from ∼35K *M. tuberculosis* clinical isolates sourced globally, which comprised the four main *M. tuberculosis* lineages (Lineage 1–4). Mutations were annotated to reflect their association with PZA resistance. Genomic measures (minor allele frequency and odds ratio), structural features (surface area, residue depth and hydrophobicity) and biophysical effects (change in stability and ligand affinity) of point mutations on pncA protein stability and ligand affinity were assessed. Missense point mutations within *pncA* were distributed throughout the gene, with the majority (>80%) of mutations with a destabilising effect on protomer stability and on ligand affinity. Active site residues involved in PZA binding were associated with multiple point mutations highlighting mutational diversity due to selection pressures at these functionally important sites. There were weak associations between genomic measures and biophysical effect of mutations. However, mutations associated with PZA resistance showed statistically significant differences between structural features (surface area and residue depth), but not hydrophobicity score for mutational sites. Most interestingly *M. tuberculosis* lineage 1 (ancient lineage) exhibited a distinct protein stability profile for mutations associated with PZA resistance, compared to modern lineages.

## Introduction

Tuberculosis (TB), is a highly infectious and contagious air-borne disease caused by the bacterium *Mycobacterium tuberculosis*. Despite its ancient origins and the efforts to develop disease control and prevention measures, the disease continues to cause a global public health burden, with increased drug resistance making control difficult. In 2019, WHO reported around 10 million global cases of TB of which 1.4 million result in death (World Health Organization [WHO], 2020). In 2019, 465,000 cases of rifampicin resistant TB (RR-TB), among which 78% cases of multidrug-resistant TB (MDR-TB, defined as having additional resistance to isoniazid) were reported. Among these RR/MDR cases, ∼6% cases were further resistant to one fluoroquinolone and one injectable second line drug, leading to extensively drug resistant TB (XDR-TB) (World Health Organization [WHO], 2020).

The size of the *M. tuberculosis* genome (reference H37Rv strain) is 4.4 Mb, with a high (65%) GC content. The *M. tuberculosis* genome is clonal, and consists of seven main lineages, which vary by their geographical spread (L1: Indo-Oceanic, L2: East Asian, L3: East-Africa-Indian, and L4: Euro-American) (Phelan et al., 2016). The lineages are further classified into ancient (L1, L5–6), modern (L2–4), and intermediate (L7) strains, with L2 being particularly mobile as evidenced by its recent spread to Europe and Africa from Asia (Phelan et al., 2016). The *M. tuberculosis* lineages appear as distinct clades on phylogenetic trees (Coll et al., 2014) and govern disease transmission and dynamics with phenotypic consequences on clinical severity and drug resistance (Ford et al., 2013; Reiling et al., 2013), including recent reports of lineage-specific associations with the latter (Oppong et al., 2019). Drug resistance in *M. tuberculosis* is almost exclusively due to mutations [including non-synonymous Single Nucleotide Polymorphisms (nsSNPs), insertions and deletions (INDELs)] in genes coding for drug-targets or drug-converting enzymes. Changes in efflux pump regulation may also have an impact on the emergence of resistance (Al-Saeedi and Al-Hajoj, 2017) and putative compensatory mechanisms have been described to overcome fitness impairment that arises during the accumulation of resistance conferring mutations (de Vos et al., 2013). Resistance-associated point mutations have been described for all first-line drugs, including rifampicin, isoniazid and pyrazinamide, as well as for several second-line and newer drugs (fluoroquinolones, bedaquiline) (Somoskovi et al., 2001; Boonaiam et al., 2010; Segala et al., 2012), but knowledge is still incomplete.

Pyrazinamide (PZA) is a crucial antibiotic used in WHO recommended combination therapies in the front-line treatment of TB. It is a pro-drug which is activated by the amidase activity of the enzyme pyrazinamidase/nicotinamidase (PZase; MtPncA) encoded by the *pncA* gene, converting PZA to its active form of pyrazinoic acid (POA). Despite its indispensable status in TB treatment, PZA’s exact mode of action remains poorly understood. Other genes (*rpsA* and *panD)* have been implicated in PZA resistance (Dookie et al., 2018) with a recent study suggesting that PZA exerts its antibacterial activity by acting as a target degrader of panD, blocking the synthesis of coenzyme A (targeted by POA) (Gopal et al., 2020). Despite this, mutations in the *pncA* gene remain the most common mechanism of PZA resistance (Khan et al., 2019).

Advances in whole genome sequencing (WGS) is assisting the profiling of *M. tuberculosis* for drug resistance, lineage determination and virulence, and presence in a transmission cluster (Phelan et al., 2019a), thereby informing clinical management and control policies. This is reflected in the WHO recommendation for use of rapid molecular testing for detecting TB and drug resistant TB (World Health Organization [WHO], 2020). The use of WGS can uncover new resistance mutations through genome-wide association studies (GWAS) and convergent evolution analysis (Phelan et al., 2016; Coll et al., 2018).

Furthermore, using protein structure, the biophysical effects of point polymorphisms can be investigated allowing a mechanistic understanding of resistance development (Phelan et al., 2016; Kavvas et al., 2018; Portelli et al., 2018). This approach can highlight important functional resistance mutations before they take hold in a population, corroborate drug susceptibility test results, as well as provide insights in highly polymorphic candidate loci (e.g., *pncA*) where many of the putative mutations have low frequency. It has been observed that sites with multiple mutations (>2) are linked to drug resistance (Comas et al., 2011), but such resistance hotspots may not necessarily lie close to the drug binding site. To this effect, sites with 2 mutations are considered as “emerging” or “budding” resistance hotspots (Portelli et al., 2018).

One assessment of the impact of missense mutations is to measure the change in a protein structure’s as well as drug-target complex’s physical interactions that contribute to its overall stability. Computational approaches (e.g., the *mCSM* suite; Pires et al., 2014a, 2016; Pires and Ascher, 2016, 2017; Rodrigues et al., 2019) have been developed to predict the effects of missense point mutations on overall protein structure stability, as well as the binding affinity/stability of ligand, protein-protein, and protein-nucleic acid interactions within a single framework, based on either an experimentally resolved structure or derived model. Here we apply such approaches to the effects of missense point mutations in the *pncA* gene. In addition, we also analyse biophysical structural features including surface area, residue depth and hydrophobicity for residues and sites associated with missense point mutations.

A crystal structure for pncA from *M. tuberculosis* has been determined as a monomeric enzyme of 186 amino acids (19.6 kDa) (Petrella et al., 2011). The structure comprises a 6-stranded parallel beta sheets, with helices on either side forming a single α/β domain with a metal cofactor (iron, Fe2+) binding site formed of D49, H51, H57, and H71. The substrate binding cavity in MtPncA is small, approximately 10 Å deep and 7 Å wide. It consists of highly conserved residues F13 and W68 that are essential in substrate binding with Y103 and H137 limiting access to this cavity (Petrella et al., 2011). The catalytic triad consisting of C138, D8, K96 is indicative of a cysteine-based catalytic mechanism (Petrella et al., 2011). Leveraging this crystal structure, we developed an *in silico* framework to assess the biophysical impact of *pncA* mutations and their resistance risk as determined by GWAS. In this study, we attempt to understand PZA resistance by exploring the relationship between the genomic features and the biophysical consequences of stability and affinity of nsSNPs, and how this is reflected in differences between *M. tuberculosis* lineages.

## Materials and Methods

### SNP Dataset

The dataset consists of 35,944 *M. tuberculosis* isolates, which has been described recently (Napier et al., 2020). In brief, it encompasses all the main lineages (1, 5, and 6, ancient; 2, 3, and 4, modern; 7 intermediate), and drug susceptibility testing across 8 first-and second-line anti-TB drugs. Across these isolates, mutations in the *pncA* coding region with non-synonymous amino acid changes (nsSNPs) were extracted. These nsSNPs were further annotated for their link with drug resistance as defined by their presence in the TB-Profiler mutation database (Phelan et al., 2019b). Initial analysis aimed at understanding the structure and characterising the active site, followed by *in silico* predictions to quantify the enthalpic and entropic effects of GWAS-identified nsSNPs on the pncA protein structure. Subsequently, additional metadata relating to the clinical isolates were studied in relation to the structural effects of mutations. The general methodology workflow followed in this analysis is similar to the one described previously (Portelli et al., 2018).

### Drug and Target: Structural Data

In the absence of a drug (PZA) and target (pncA) complex, respective individual structures were obtained from RSCB PDB database (Berman et al., 2000). The crystal structure of *pncA* in *M. tuberculosis* is available as PDB entry 3PL1 (Petrella et al., 2011), while the structure of PZA was extracted from PDB entry 3R55 (Singh et al., 2011). The molecular motion of pncA was analysed by Normal Mode Analysis using the DynaMut tool (Rodrigues et al., 2018) (Supplementary Figure 1).

### Protein-Ligand Docking: Autodock Vina

The *pncA*-PZA complex was generated using the software AutoDock Vina, version 1.1.2 (Trott and Olson, 2009). Autodock Vina is an open-source, freely available molecular modelling platform to perform protein-ligand docking. Docking was carried out with default settings and guided by the positioning of the ligand within the active site as descried by Petrella et al. (2011). The complex was generated to facilitate downstream analyses by mCSM-lig (Pires et al., 2016) Autodock Vina returns bound conformations with their respective predicted binding affinity values. The prediction of binding affinity (strength of the ligand interaction with its target) is based on one of several scoring functions, which rank the poses in increasing order of predicted binding affinity. Binding free energy is calculated using a semi-empirical force field, combining experimental and knowledge-based information. The docking poses were visualised and inspected in UCSF Chimera 1.13 (Pettersen et al., 2004) according to the occupation of search space and diversity of pose conformations (Supplementary Figure 2). The top two binding poses were closely matched with the conformations generated by Karmakar et al. (2018) and Petrella et al. (2011), respectively (Supplementary Figure 3). The best pose was chosen considering the ligand orientation generated by molecular docking performed by Karmakar et al. (2018) and comparing interaction of both poses with active site residues through an Arpeggio (Jubb et al., 2017) analysis (Supplementary Figure 4).

Ligand extraction and protonation were carried out using UCSF Chimera, version 1.11 (Pettersen et al., 2004) while identification of rotatable bonds was carried out in Autodock tools (available as part of MGL tools, version 1.5.6) (Morris et al., 2009) where protonation of the ligand is specifically required by Autodock Vina (Trott and Olson, 2009). Similarly, protein extraction and explicit removal of solvent were carried out in UCSF Chimera, version 1.11 (Pettersen et al., 2004), and other steps in the overall protein preparation process were carried out in Autodock tools (part of MGL tools, version 1.5.6) (Morris et al., 2009). All the required parameters to perform docking needed to be included in a configuration file.

### *In silico* Predictions: mCSM DUET, FoldX, mCSM-lig

The computational tools based on mutation cut-off scanning matrix, primarily *mCSM DUET* (Pires et al., 2014a) and *mCSM-lig* (Pires et al., 2016) were used to investigate the structural effects of nsSNPs within the pncA target protein. The effects of nsSNPs within *pncA* were analysed with respect to protein stability (DUET and FoldX (Schymkowitz et al., 2005) and ligand affinity (mCSM-lig). The consequences of these effects were to investigate change in protein fold and function, and effect on mechanism of PZA drug activation, respectively. Results from mCSM-lig (Pires et al., 2016) return both ligand affinity and DUET scores, hence only mCSM-lig was run to obtain both the outputs simultaneously.

A semi-automated pipeline was constructed for mCSM and FoldX to submit and extract results for multiple mutations consecutively using python and shell scripts. Both tools require wild type structure, chain ID and a list of nsSNPs in the X <*POS*> Y format (X: wild type residue; <*POS*> : position, Y: mutant residue). The residue symbols (X and Y) are specified as one letter amino acid code. DUET and FoldX estimate mutational impact as a change in Gibbs Free energy (ΔΔG) in Kcal/mol. The classification of mutational impact based on ΔΔG from these methods are categorised in opposing ways. For example, ΔΔG < 0 of a SNP is classified as a “destabilising” according to DUET, while the same is classified as “stabilising” according to FoldX.

The mutational impact on ligand affinity is calculated as a log fold change between wild type and mutant binding affinities. In addition to SNP identifiers, mCSM-lig requires the ligand affinity of the wild-type protein to be specified in nano Molar (nM) for affinity change calculations. Since the binding affinity returned by AutoDock Vina, version 1.1.2 (Trott and Olson, 2009) is in Kcal/mol, these needed to be converted to nM via Eq. 1 (below). The binding affinity for PZA in nM was 0.9911.

(1) $$\begin{array}{c} \Delta G=-\mathrm{RTlnK}. \end{array}$$

*Equation 1:* Calculation of binding free energy, ΔG, where R is the gas constant, 1.987 cal K^–1^ mol^–1^ and T is the absolute temperature, 298 K. Adapted from Morris et al. (1998).

The mCSM suite of tools (Pires et al., 2014a, 2016; Pires and Ascher, 2017; Rodrigues et al., 2019) are based on graph-based measures at an atomic level along with machine learning (ML) tools for predicting enthalpic and entropic effects of stability. mCSM achieves this broadly by generating a signature encompassing the wild-type milieu and change in pharmacophore properties upon mutation (Pires et al., 2014b). Owing to the inter-atomic distance pattern within mCSM describing the wild-type residue environment, novel parameters like residue depth and long-range interactions are implicitly considered. In this manner, mCSM is able to characterise both local and global effects of missense point mutations. The mutational change at the atomic level is considered by using a change in the “pharmacophore count” vector, thus obviating the need to have explicit mutant structure. All mCSM tools (Pires et al., 2014a, 2016; Pires and Ascher, 2016, 2017; Rodrigues et al., 2019) use the atomic changes, while DUET (Pires et al., 2014a) is an ensemble method combining methods of mCSM stability (Pires et al., 2014b) and SDM (Worth et al., 2011; Pandurangan et al., 2017). FoldX, however is an empirical-based prediction tool which summarises the change in stability between mutant and wild type protein structures using a combination of energy terms based on fundamental intramolecular interactions (Schymkowitz et al., 2005).

### Other Structural Parameters

Additional structural parameters for wild type structure were also included in the analysis. These were: Accessible (ASA) and Relative Surface Area (RSA), residue depth (RD), hydrophobicity values according to the Kyte-Doolittle scale (KD). The DSSP programme (Kabsch and Sander, 1983; Touw et al., 2015) was run to extract the ASA and RSA values, while RD values calculated as described by Chakravarty and Varadarajan (1999) were calculated using the depth server available at http://cospi.iiserpune.ac.in/depth. The KD values were fetched from the expasy server (Artimo et al., 2012) available at https://web.expasy.org/protscale/.

### Data Normalisation: DUET, FoldX, and mCSM-lig

The DUET (Pires et al., 2014a), FoldX (Schymkowitz et al., 2005), and mCSM-lig (Pires et al., 2016) scores associated with each SNP were subsequently normalised between the range of −1 and 1. For mCSM-lig analyses, data was filtered according to distance from interacting site and only residues within a distance of 10 Å of the ligand (PZA) were considered for all ligand affinity analyses.

### Minor Allele Frequency and Odds Ratio Calculations: SNP Dataset

Across the *M. tuberculosis* isolates tested for PZA drug susceptibility data, we performed association analysis to estimate the risk of resistance for SNP alleles. For each nsSNP, minor allele frequency (MAF) and odds ratio (OR) were calculated in relation to all samples tested for PZA susceptibility. MAF is the average occurrence of a given nsSNP, and OR is the measure of association of a given nsSNP with PZA resistance. In addition to unadjusted odds ratio (OR), and similar to a GWAS approach, adjusted odds ratio (aOR) were estimated using logistic regression models with a kinship matrix adjusting for a random effect representing the SNP-based relationships between samples (e.g., the lineage-based population structure) (Zhou and Stephens, 2012; Coll et al., 2018). *P*-values were estimated using Fisher and Wald test for unadjusted and adjusted ORs, respectively.

### Statistical Analyses

Data was analysed using non-parametric statistical tests. For assessing correlations, Spearman correlation values were calculated. For comparing lineage distributions, the Kolmogorov-Smirnov (KS) test was used. Statistical significance thresholds used are ^∗^*P* < 0.05, ^∗∗^*P* < 0.01, ^∗∗∗^*P* < 0.001, ^****^P < 0.0001).

### Data Visualisation

All plots were generated using R statistical software, version 4.0.2 (R Core Team, 2014). Protein and ligand structures were generated using UCSF Chimera, version 1.11 (Pettersen et al., 2004).

## Results

### Analysing the pncA Molecular Motion and pncA-PZA Complex

**FIGURE 1:**
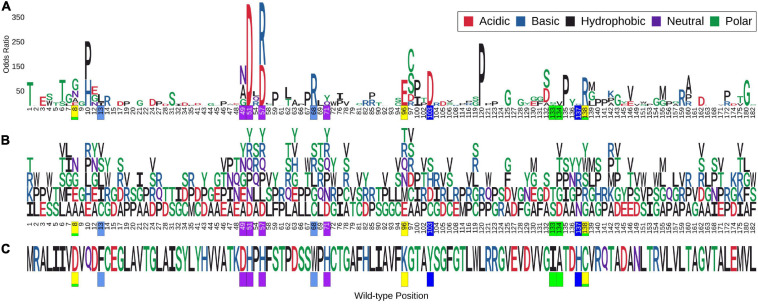
Logo plot showing sites with multiple missense point mutations and association with Odds Ratio. Sites associated with multiple (>2) missense point mutations (i.e., nsSNPs). A total of 386 mutations corresponding to 113 positions on the pncA protein structure were associated with multiple nsSNPs. The horizontal axis in **(A,B)** show the position numbers of sites with multiple nsSNPs, while part **(C)** shows the wild-type residues for each position. The vertical axis in **(A)** represents Odds Ratio (OR) where letters denote mutant residues which are proportional to their corresponding OR highlighting the most resistant mutation at each site and overall. Part **(B)** shows each mutant residue at a given position, highlighting nsSNP diversity by position. The wild-type and mutant residues are coloured according to the amino acid properties as denoted. Positions marked in yellow form the catalytic triad, residues in blue and teal are involved in substrate binding, those in green are involved in hydrogen binding while the ones in purple are involved in the iron centre coordination. The figure is generated using R statistical software (version 4.0.2). nsSNPs, non-synonymous Single Nucleotide Polymorphisms; pncA, pyrazinamidase.

Molecular motion in pncA was analysed by Normal Mode Analysis (NMA). Regions undergoing the greatest movement were limited to residues in loop regions and mainly concentrated to loop 60–66, followed by loop residues 39–41 and 111–113. Residues at site 165–167 within helix 164–178 showed the least flexibility (Supplementary Figure 1). The frequency of mutations in these variable regions was most prominent for sites 62–63 (>2 mutations) while the other sites were limited to at most two mutations (Figure 1). Mutations within the most flexible region (residues 60–66) of pncA showed mixed effects in relation to their association with PZA resistance with the single mutation at site 64 related to PZA resistance. Sites 39 and 40 within the other highly flexible region 39–41 were not associated with any mutations in our study, while the two mutations at site 41 were not associated with PZA resistance. The region 111–113 is associated with single mutations at sites 111 and 112 which are not linked to PZA resistance, while site 113 was not associated with any mutations in our study. Sites 165–167, which form part of the helix (164–178), are the most stable according to NMA. Two residues (A165 and D166) within this helix were not associated with any mutations in our study, while a single mutation at site T167 was not associated with PZA drug resistance (Supplementary Figure 1 and Supplementary Table 1). Docking with AutoDock vina (Trott and Olson, 2009) generated nine different conformations as per default settings. In six of these poses, the aromatic ring of PZA was oriented towards the substrate binding residue W68 (Supplementary Figures 2A,B). The top two poses (1 and 2) returned by Vina were similar to previous molecular docking studies (Petrella et al., 2011; Karmakar et al., 2018) (Supplementary Figure 3). A follow-up Arpeggio analysis (Jubb et al., 2017) indicated that pose 1 when compared to pose 2, has more H-bonds (4 vs. 1), fewer aromatic contacts (3 vs. 13), and greater Van der Waals interactions (3 vs. 1) (Supplementary Figures 4A,B). Therefore, model with pose 1 was chosen to form the pncA-PZA complex (Supplementary Figure 5).

### Genomics Data

SNP data from 35,944 *M. tuberculosis* clinical isolates tested for drug susceptibility to a range of first and second line drugs were obtained (Napier et al., 2020). Among these, 39% (*n* = 13,914) of these isolates were tested for PZA drug susceptibility. The isolates were collected from over 30 different countries and represented the 4 main *M. tuberculosis* lineages (L1, *n* = 144; L2, *n* = 1,886; L3, *n* = 190; L4, *n* = 2213) (Supplementary Figure 6). In order to infer whether the ancestral pncA sequences for each lineage differed, we quantified the number of samples without any mutations in each lineage. The majority of isolates in L1–L4 had an identical *pncA* sequence as the H37Rv reference indicating that the ancestral sequences for these lineages do not differ. The majority were pan susceptible (*n* = 23,256, 64.7%), with the remainder MDR-TB (*n* = 6,691, 18.6%), XDR-TB (*n* = 989, 2.8%), or another type of resistance referred to as DR-TB (*n* = 5,008, 13.9%) (Table 1). From the list, only nsSNPs within the protein coding region of *pncA* (*n* = 4,731, 13.2%) were considered for our analyses (Table 1). The majority of these were MDR-TB (*n* = 3,290, 69.5%) followed by relatively equal numbers of XDR-TB and DR-TB (*n* = 625, 13.2% and *n* = 632, 13.4%, respectively), while only a small percentage were susceptible (*n* = 184, 3.9%) (Table 1). From a total of 13,914 samples tested for PZA drug susceptibility, a minority of those were found to be resistant (*n* = 2,379, 17.1%) (Table 1). However, the burden of PZA resistance among samples containing nsSNPs in the protein coding region was high (*n* = 1,677, 73.3%) (Table 1).

**TABLE 1:** Number of samples analysed.

Item name	Total number (%)
Clinical isolates/samples	35,944
Samples classified Susceptible	23,256 (64.7)
Drug resistant (DR)	5,008 (13.9)
Multi-drug resistant (MDR)	6,691 (18.6)
Extreme drug resistant (XDR)	989 (2.8)
Samples tested for PZA drug susceptibility	13,914
Resistant	2,379 (17.1)
Samples with nsSNPs in the protein coding region of *pncA*	4,731 (13.2)
Susceptible	184 (3.9)
Drug resistant (DR)	632 (13.4)
Multi-drug resistant (MDR)	3,290 (69.5)
Extreme drug resistant (XDR)	625 (13.2)
Samples with *pncA* nsSNPs tested for PZA drug susceptibility	2,289 (48.4)
Samples with *pncA* nsSNPs resistant to PZA	1,677 (73.3)
Unique nsSNPs: No. of sites	424 nsSNPs: 151 sites

*Summary of clinical isolates from genome-wide analysis. PZA, pyrazinamide; nsSNPs, non-synonymous Single Nucleotide Polymorphisms.*

**FIGURE 2:**
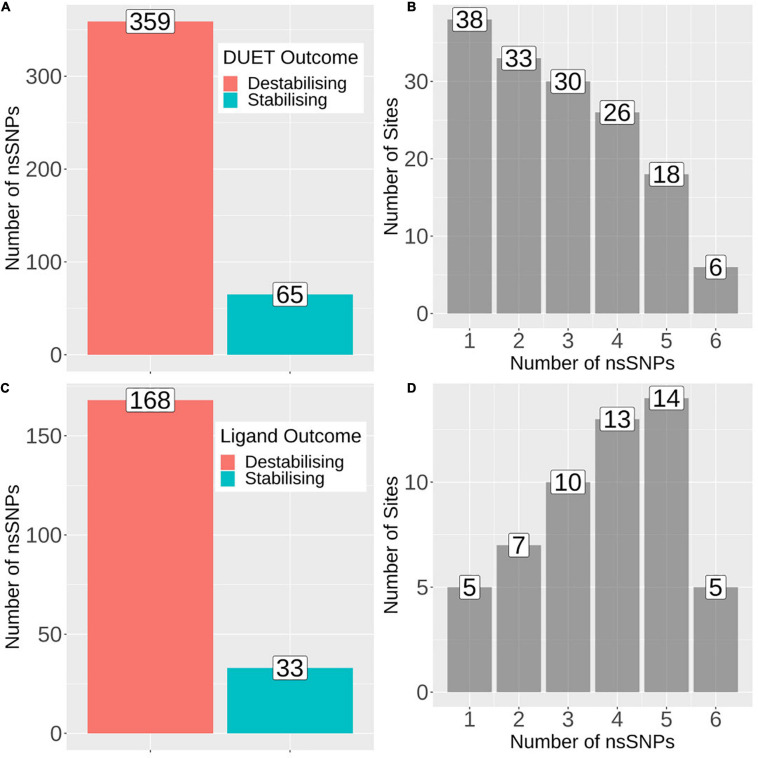
Barplots showing number of mutations and sites associated with protein stability and ligand affinity. **(A)** Number of nsSNPs categorised as destabilising (*n* = 359) and stabilising (*n* = 65) according to DUET protein stability. **(B)** Frequency of sites associated with the number of nsSNPs, where horizontal axis denotes the number of nsSNPs and vertical axis denotes the total number of sites/positions corresponding to the number of nsSNPs. **(C)** Barplot showing the number of nsSNPs categorised as destabilising (*n* = 168) and stabilising (*n* = 33) according to mCSM ligand affinity where sites lie within 10Å of ligand. **(D)** Frequency of sites associated with the number of nsSNPs, where horizontal axis denotes the number of nsSNPs and vertical axis denotes the total number of sites/positions corresponding to the number of nsSNPs. The figure is generated using R statistical software (version 4.0.2). nsSNPs, non-synonymous Single Nucleotide Polymorphisms.

Across the 4,731 isolates, 424 distinct nsSNPs corresponding to 151 distinct amino acid positions on the pncA structure were identified (Figures 2A,B). A total of 201 nsSNPs corresponding to 54 amino acid changes were within 10 Å of the ligand binding site (Figures 2C,D). The majority of these nsSNP mutations have been annotated as being linked to PZA resistance within the TBProfiler tool (227/424). The majority of these nsSNP mutations have been annotated as being linked to PZA resistance within the TBProfiler tool (227/424; denoted as DM), while the others (197/424; denoted as OM) were assumed to have weak or no links. Genomic measures like minor allele frequency (MAF) and odds ratio (OR) were obtained for a total of 322 nsSNPs, with adjusted OR (aOR) estimated for a total of 163 nsSNPs. Across the majority of these nsSNPs, the MAFs were low (median: 0.02% range: 0.01–2.11%) (Supplementary Figure 7A). Similarly, when considering ORs, the majority of the nsSNPs had high ORs (median: 9.70, range: 0.22–414.61) (Supplementary Figure 7D). When looking at the distribution of MAF and OR within mutations associated with PZA resistance (DM) and other mutations (OM) (Supplementary Figures 7B,E), DM mutations were associated with significantly higher (*P* < 0.0001) MAF and OR (Supplementary Figures 7C,F).

### Understanding Mutational Effects on pncA Stability and PZA Binding Affinity

**FIGURE 3:**
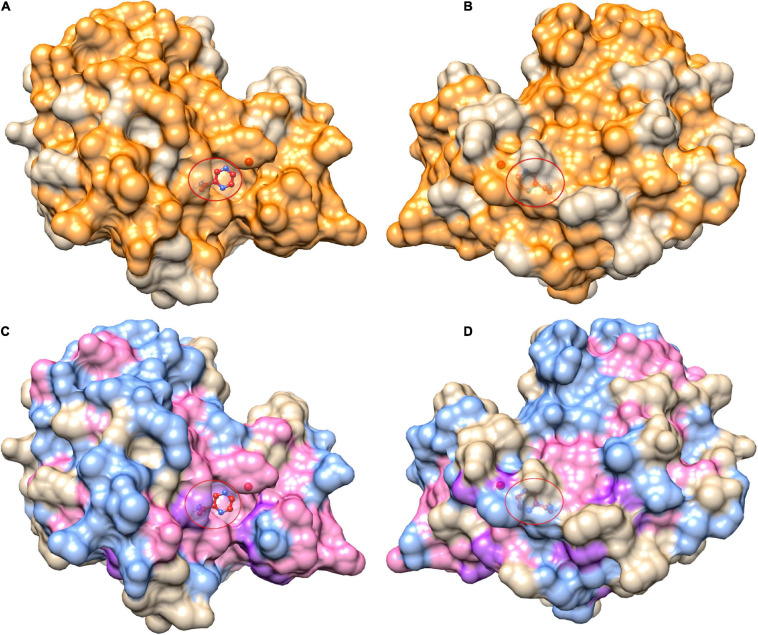
Mutational landscape of pncA structure (3PL1) coloured by positions linked to pyrazinamide drug (PZA) resistance. Panels **(A,B)** show all mutational positions in orange while mutational positions in **(C,D)** are further coloured by mutations classed as either drug resistant mutations (purple) or “other mutations” (blue), while sites linked to mutations belonging to either category are coloured in pink. The right panels **(B,D)** depict the corresponding structure rotated by 180°. The ligand (PZA) is shown as ball and stick within the active site denoted by the red circle. The figure is rendered using UCSF Chimera (version 1.14). pncA, pyrazinamidase.

**FIGURE 4:**
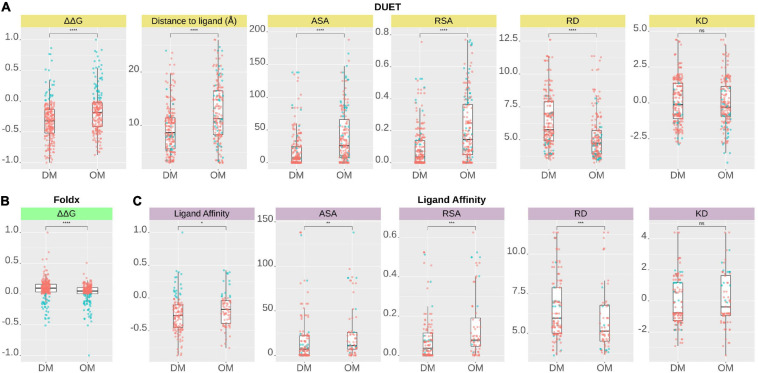
Comparison of structural features between Drug resistance (DM) and other mutations (OM) of pncA gene mutations according to **(A)** DUET protein stability (ΔΔG), **(B)** FoldX stability (ΔΔG), and **(C)** Ligand Affinity. A total of 424 nsSNPs for DUET and FoldX (DM, *n* = 227, OM, *n* = 197), while a total of 201 nsSNPs (DM, *n* = 129 OM, *n* = 72) lying within 10 Å of PZA for ligand affinity were included in the analysis. DM and OM mutations were compared using Wilcoxon rank-sum (unpaired) and statistical significance indicated as: *P < 0.05, **P < 0.01, ***P < 0.001, ****P < 0.0001). The figure is generated using R statistical software (version 4.0.2). ns, non-synonymous Single Nucleotide Polymorphisms; pnca, pyrazinamidase; PZA, pyrazinamide; Å, Angstroms; ΔΔG, Change in Gibbs free energy in Kcal/mol; ASA, Accessible Surface Area; RSA, Relative surface Area; RD, Residue Depth; KD, Kyte-Doolittle Hydrophobicity values.

**FIGURE 5:**
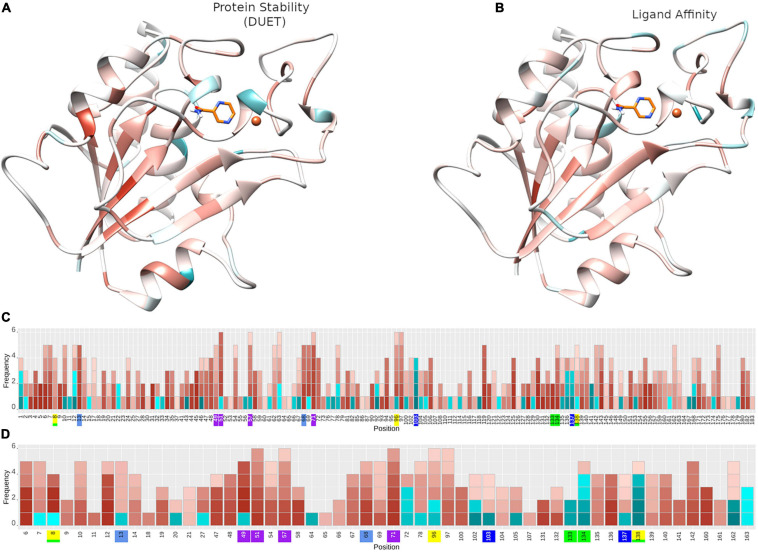
Protein stability and ligand affinity effects of nsSNPs on pncA structure and by position. Mutational impact of nsSNPs on the pncA protein structure coloured by average **(A)** DUET Protein stability (*n* = 424) and **(B)** ligand affinity (*n* = 201). Barplots **(C,D)** showing the frequency of mutations within the pncA gene. The horizontal axis shows the mutational positions within pncA and the vertical axis shows the frequency of mutations. Positions on the horizontal axis are coloured to denote the active site residues: green (residues involved in hydrogen bonding with PZA), yellow (catalytic triad), blue and teal (substrate binding), purple (iron centre). For a given position, each corresponding mutation (nsSNP) is coloured by the level of stability according to **(C)** DUET(*n* = 424) and **(D)** Ligand affinity (*n* = 201) where the horizontal axis denotes amino acid positions in pnca, and is restricted to positions lying within 10 Å of PZA for ligand affinity. Destabilising mutations are depicted in red and stabilising mutations in blue, where colour intensity reflects the extent of effect, ranging from −1 (most destabilising) to + 1 (most stabilising). The structural figures **(A,B)** are rendered using UCSF Chimera (version 1.14). The barplot figure **(C,D)** is generated using R statistical software (version 4.0.2). nsSNPs, non-synonymous Single Nucleotide Polymorphisms; PZA, pyrazinamide; pncA, pyrazinamidase.

The 424 nsSNPs mapped onto the crystal structure of pncA revealed that mutational landscape of pncA appears distributed (Figures 3A,B) throughout the structure. Sites linked to drug resistant mutations were predominant around the PZA binding (active) site, while sites exclusively linked to mutations classed in the “other” category are distal to the active site (Figures 3C,D, 4). Furthermore, active site residues were associated with a multiple point mutation (Table 2 and Figures 1B, 5C). All active site and hydrogen-bond forming residues with the ligand were associated with multiple mutations (≥2) (Figure 1B), thus representing the high diversity of mutations present within pncA. Despite this, there appears to be some degree of clustering around positions 4–14, 46–97, 132–143 involving the active site and metal centre residues (Figure 5C).

**TABLE 2:** Mutations close to the active site of PZA.

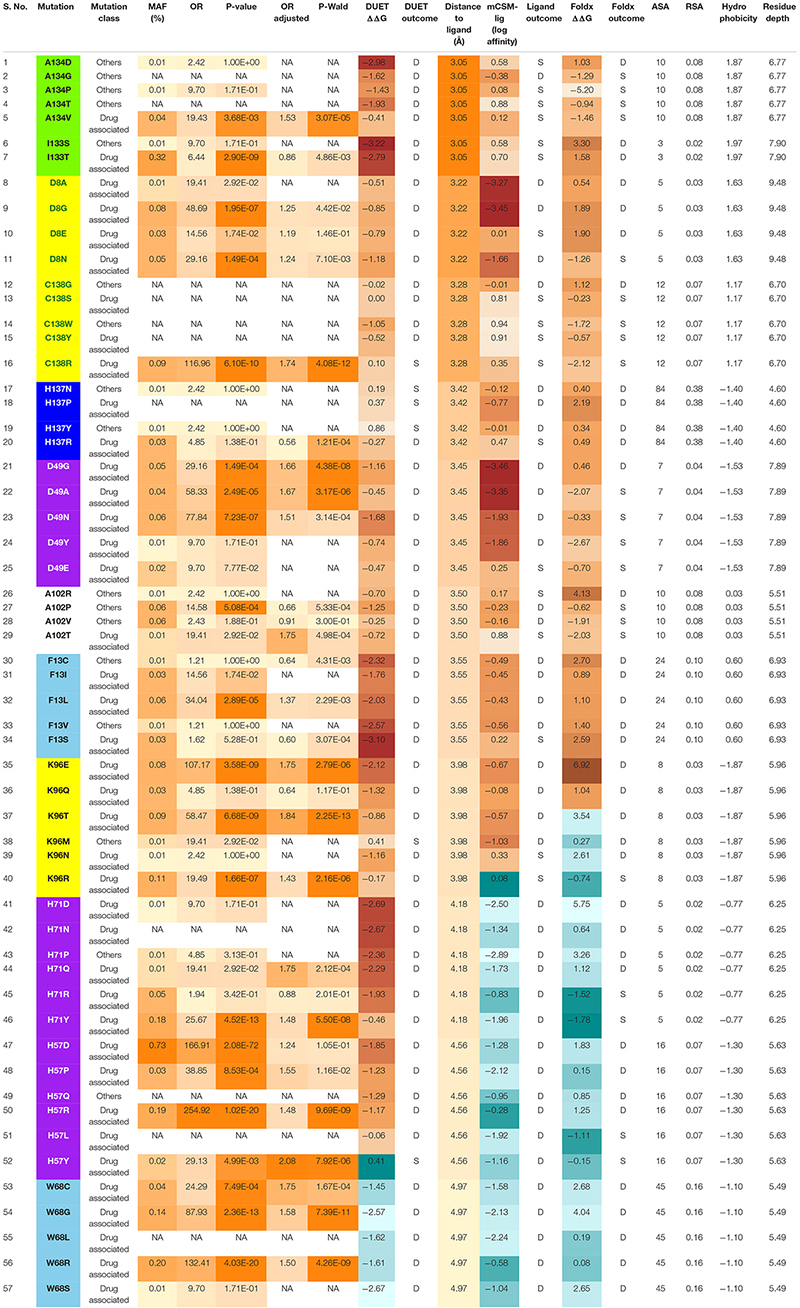

*Fifty-seven mutations (nsSNPs) lying within 5 Å of PZA and the corresponding GWAS measures of minor allele frequency (MAF), Odds Ratio (OR), P-values, adjusted OR (aOR), and P-values from Wald test corresponding to aORs, along with structural measures of distance to ligand, DUET, FoldX, ligand affinity values and effect. Wild type residues for mutations highlighted and marked in green are considered to participate in hydrogen bonding, those in yellow form the catalytic triad, residues in teal (and blue) are involved in substrate binding, while the residues in purple are involved in the iron centre. The columns are coloured to highlight the most significant column attribute with deeper colours denoting the greatest effects. The dark colours in MAF, OR, and aOR columns indicate the highest values, while P-values are coloured with the darkest colour showing the most significant values. Values in the DUET, mCSM-lig, and FoldX columns are coloured according to the extent of their respective effects with red indicating destabilising and blue denoting stabilising effects. nsSNPs, non-synonymous Single Nucleotide Polymorphisms; PZA, pyrazinamide; GWAS, Genome-Wide Association Studies. D, Destabilising; S, Stabilising.*

The biophysical effect of mutations on protomer stability, estimated as ΔΔG (Kcal/mol), was measured using DUET (Pires et al., 2014a) and FoldX (Schymkowitz et al., 2005), while mutational impact on ligand affinity was measured using mCSM-lig (Pires et al., 2016) (see section “Materials and Methods”). Assessing mutational effects on protein stability as measured by DUET, nearly 85% had a destabilising effect (*n* = 359) compared to nearly 15% mutations with stabilising effects (*n* = 47) as shown in Figure 2A. When assessing ligand affinity, 47.4% (*n* = 201) SNP mutations were present within 10 Å of the PZA binding site (Figure 2C). Similar to DUET stability effects, the majority (84%; *n* = 168) of nsSNPs were destabilising while 16% (*n* = 27) were stabilising for ligand binding affinity (Figure 2C). More than 50% of the mutational positions were associated with multiple nsSNPs for both protein stability (*n* = 113) and ligand affinity (*n* = 49) (Figures 2B,D). The average protein stability and ligand affinity effects of all mutations mapped onto the pncA structure (Figures 5A,B), highlight mutations with opposing effects for protein stability and ligand affinity. These effects are pronounced for active site residues (I133, A134, H137, C138) (Figures 5C,D).

There were 80 sites within *pncA a*ssociated with multiple nsSNPs (>2) (Figures 1B, 2B) which included all active residues except I133 which was associated with 2 mutations (Figure 1B). Sites with 2 nsSNPs are considered to be budding resistance hotspots (*n* = 33 for protein stability, *n* = 7 for ligand affinity). A total of 57 nsSNPs within 5 Å of PZA were considered to be within the first shell of residues lining the active site (Table 2). While majority of the mutational sites associated with more than two mutations comprise of destabilising mutations, positions 1, 2, 10, 12, 43, 46, 51, 57, 63, 67, 69, 78, 82, 92, 96, 100, 104, 105, 129, 135–138, 142, 149, 164, 168, and 174 comprised of both stabilising and destabilising mutations (Figure 5C). Similarly, for ligand affinity, most mutational sites had destabilising mutational effects, with positions 7, 8, 13, 27, 49, 72, 78, 96, 102, 103, 105, 134, 137, 138, and 162 associated with mutations resulting in mixed stability impact. Position 163 comprised only of mutations with stabilising effects (Figure 5D). The budding resistance hotspot active site residue I133 contained both mutations with destabilising effect for protein stability (Figure 5C), while stabilising for ligand affinity (Figure 5D). Similarly, for budding resistance hotspots, majority of the nsSNPs were associated with destabilising effects. For protein stability, 9/33 sites had mutations with mixed stability (positions 15, 32, 61, 66, 76, 114, 127, 153, and 161) (Figure 5C), while only position 20 showed mixed stability effects for ligand affinity (Figure 5D).

### Mutations With Extreme Effects

Mutations with extreme effects on protein stability and affinity are summarised in Table 3. Overall, the most destabilising mutation according to DUET was L4S, where a change from a hydrophobic to a polar residue may contribute to disruption of local conformation (Table 3). The closest most destabilising mutational effect on protein stability was from A134D (wild-type residue involved in hydrogen bonding) (Table 3), likely resulting in electrostatic and steric clashes due to a change in charge and volume affecting the overall stability negatively. The most stabilising mutation on protomer stability was from active site residue Y103D, while the closest such mutation was C138R (Table 3). The stabilising effect of these mutations on the protein stability and ligand affinity is thought to result from the electrostatic interactions working favourably for sites lying within 5 Å of the ligand. The most destabilising mutation according to ligand affinity was D49G located at ∼3.5 Å (Table 3). The three subsequent destabilising mutations for ligand affinity were also all within 5 Å of PZA binding site namely D8G (∼3 Å), D49A (∼3.5 Å), and D8A (∼3 Å) (Supplementary Table 1), all arising likely due to the loss of charge and volume interfering with ligand interaction. The mutation with the greatest stabilising effect on ligand affinity was G162D, located at ∼8 Å, i.e. outside the first shell of influence (>5 Å) from the ligand. This is possibly due to the resulting electrostatic effects and increase in volume, which may favour hydrogen bond formation with nearby residues and PZA binding, thereby increasing affinity (Table 3). The closest most stabilising mutational impact on ligand affinity was due to mutation A134P, though this was a marginal effect (Table 3). The most destabilising mutation according to FoldX was C72W, which is located far away from the active site (∼27 Å). Interestingly, mutation A134P was the most stabilising according to FoldX, while the same was estimated to have a destabilising effect according to DUET (Table 3). All mutations except A134D and A134P were associated with PZA drug resistance (Table 3).

**TABLE 3:** Mutations with extreme effects.

Mutational effects	Mutation	Mutation class	MAF (%)	OR	*P*-value	Distance to ligand (Å)	Stability ΔΔG	Ligand affinity
Highest OR	H51D	Drug-associated	0.30	414.61	4.49E-33	5.66	−2.2	−1.82
Most frequent mutation	Q10P	Drug-associated	2.11	156.23	1.28E-207	6.02	−0.63	−1.77
Most deStabilising for protein stability (DUET)	L4S	Drug-associated	0.25	28.46	5.63E-18	15.33	−3.87	−1.08
Closest destabilising for protein stability (DUET)	A134D	Others	0.007	2.43	1.00	3.05	−2.98	0.58
Most stabilising for protein stability (DUET)	Y103D	Others	0.22	142.33	1.24E-21	5.42	1.18	0.85
Closest stabilising for protein stability (DUET)	C138R	Drug-associated	0.09	116.96	6.09E-10	3.28	0.10	0.35
Most destabilising for ligand affinity	D49G	Drug-associated	0.05	29.16	0.0001	3.45	−1.16	−3.46
Closest destabilising for ligand affinity	D8G	Drug-associated	0.08	48.69	1.95E-07	3.22	−0.85	−3.45
Most stabilising for ligand affinity	G162D	Drug-associated	0.03	38.85	0.0008	8.32	−1.04	2.23
Closest stabilising for ligand affinity	A134P	Others	0.007	9.70	1.71E-01	3.05	−1.43	0.08
Most destabilising for protein stability (Foldx)	C72W	Drug-associated	0.01	19.41	0.03	7.05	27.46	–
Most stabilising for protein stability (Foldx)	A134P	Others	0.007	9.70	1.71E-01	3.05	−5.2	–

*Mutations (nsSNPs) with extreme effects on odds ratio, frequency, thermodynamic stability, and ligand affinity. For ligand affinity, only mutations lying within 10 Å of PZA (pyrazinamide) were considered. nsSNPs, non-synonymous Single Nucleotide Polymorphisms; Å, Angstroms; MAF, minor allele frequency; OR, Odds Ratio; ΔΔG, Change in Gibbs free energy in Kcal/mol.*

### Relating Structural and GWAS Analyses

The minor allele frequencies for the 424 nsSNPs were mapped onto their corresponding amino acid positions of the *pncA* gene (Supplementary Figure 8). Position 10 had the highest cumulative minor allele frequency (MAF, ∼2.3%), followed by position 7 (∼1.2%), position 57 (∼1.0%), position 51 (∼0.6%), and position 14 (0.5%). The risk of PZA resistance from the alleles at each SNP was estimated by calculating ORs and *P*-values using a GWAS approach. Additionally, adjusted OR (aOR) which accounted for the confounding effects of lineage were also analysed (Supplementary Figure 9). The majority of nsSNPs were linked to increased likelihood of being resistant to PZA (OR > 1). For unadjusted ORs, this was 96% (310/322), while for aOR, it was ∼75% (122/163). Wild type position 51 had the highest unadjusted OR (> 350, *P* < 10^–30^), followed by positions 57, 120 (OR > 250, *P* < 10^–19^), and subsequently by positions 10, 103, 68, 135, 138, 96, and 180 (OR > 100; *P* < 10^–10^) (Figure 1A, Supplementary Figure 8, and Supplementary Table 1), with most of these positions being present in the metal binding and active sites.

When assessing sites in relation to mutational diversity, active site residues were among the highest, with residues H51, H57, H71, K96 associated with six distinct mutations, followed by F13, D49, W68, A134, C138 with five mutation each, while residues D8, Y103, H137 were associated with four distinct mutations and residues I133 associated with two distinct mutations (Figure 1B). The dominant effect of a highly frequent mutation (Q10P; MAF = 2.1%, *OR* = 156.23) in the population compared to two other mutations observed at the same position namely Q10R (MAF = 0.13%, *OR* = 83.01) and Q10H (MAF = 0.08%, *OR* = 107.17) (Supplementary Table 1), makes position 10 prominent in terms of MAF (Supplementary Figure 8) while sites involved in the catalytic activity and iron metal centre are more prominent with respect to SNP diversity (Supplementary Figure 8). These results suggest that mutations at these structurally and functionally important sites are likely under selective pressure exerted by the drug resulting in this observed mutational diversity.

**FIGURE 6:**
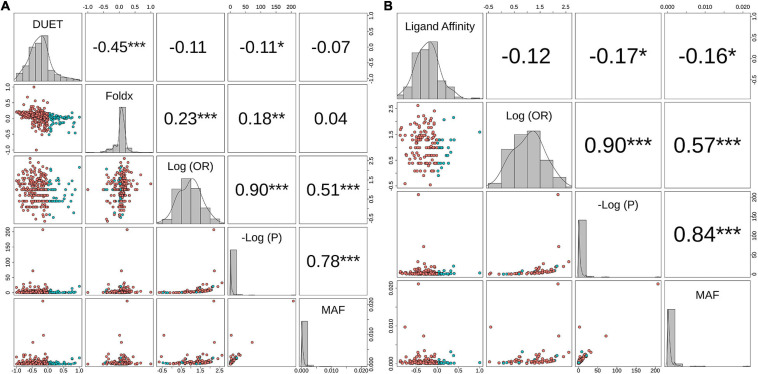
Correlation between biophysical effects and GWAS measures of Odds Ratio (OR), *P*-values (P) and minor allele frequency (MAF). Pairwise correlations between MAF, negative log10 *P*-value [-Log(P)], Log10 (OR) and **(A)** Protein stability (DUET) and FoldX for 424 nsSNPs, **(B)** Ligand affinity of 201 nsSNPs (lying within 10 Å of PZA). The upper panel in both plots include the pairwise Spearman correlation values along with their statistical significance (*P < 0.05, **P < 0.01, ***P < 0.001). The points in the lower panel represent nsSNPs, coloured according to respective stability effects: **(A)** nsSNPs with destabilising effect for DUET and ligand affinity are coloured red, while for FoldX these appear in blue, **(B)** nsSNPs with stabilising effect for DUET and ligand affinity appear in blue, while for FoldX these appear in red. The diagonal plots display the histogram of the corresponding parameter. The figure is generated using R statistical software (version 4.0.2). nsSNPs, non-synonymous Single Nucleotide Polymorphisms; PZA, pyrazinamide; Units for DUET, FoldX and Ligand Affinity (Kcal/mol).

The relationship between structural measures of stability and OR was visualised as a bubble plot indicating that mutations associated with greater resistance (high OR) tend not to have extreme effects (Supplementary Figure 10). Furthermore, this relationship along with MAF, OR, and *P*-values was assessed through Spearman correlations (Figures 6A,B). MAF was strongly correlated with *P*-values for all 424 mutations (ρ = 0.78, *P* < 0.001) and 201 mutations lying with 10 Å of PZA (ρ = 0.84, *P* < 0.001) (Figures 6A,B). As expected, OR and *P*-values were strongly correlated (ρ = 0.9, *P* < 0.001) for all 424 nsSNPs and 201 nsSNPs close to PZA binding site (Figures 6A,B). FoldX stability and DUET stability values showed moderate correlation (ρ = 0.45, *P* < 0.001). The negative sign for the DUET and FoldX associations is expected since stability changes measured by these tools have opposite signs (i.e., ΔΔG < 0: destabilising in DUET vs. stabilising in FoldX). FoldX ΔΔG values showed weak but significant correlations with OR (ρ = 0.23, *P* < 0.001), and *P*-values (ρ = 0.18, *P* < 0.01) (Figure 6A), while DUET ΔΔG and ligand affinity showed weak and insignificant association with OR (ρ = −0.1, *P* > 0.05) (Figures 1B, 6A), including adjusted OR (Supplementary Figures 9A, 8B).

When considering aOR and its relationship with stability and other structural features [i.e., Accessible (ASA), Relative Surface Area (RSA), residue depth (RD), and hydrophobicity values (KD)], there was high correlation (ρ > 0.6, *P* < 0.05) with adjusted and unadjusted ORs (Supplementary Figure 9A). DUET ΔΔG showed moderate positive correlation between ASA and RSA (ρ > 0.6, *P* < 0.05), while moderately negative correlation with RD (ρ∼−0.5, *P* < 0.05), and weak negative correlation with KD values (ρ∼−0.2, *P* < 0.05) (Supplementary Figure 9A). The same structural features, however, did not demonstrate correlation with either FoldX ΔΔG (Supplementary Figure 9A) or ligand affinity (Supplementary Figure 9B).

### Structural Differences in Drug Associated Mutations

Comparing stability effect (DUET and FoldX), ligand affinity, ligand distance, and other structural features (ASA, RSA, RD, KD) between mutations associated with PZA drug resistance (DM) and other mutations (OM), revealed statistically significant differences (*P* < 0.05) between all features except hydrophobicity values. The difference in structural features were most prominent when all 424 SNP mutations were considered (*P* < 0.0001) (Figures 4A,B) with lesser significance for ligand affinity (*P* < 0.05), ASA (*P* < 0.01), and RSA and RD (*P* < 0.001) values when 201 nsSNPs lying within 10 Å were considered (Figure 4C). Mutations associated with PZA resistance have lower DUET (Figure 4A, top left) but higher FoldX stability changes (Figure 4B, bottom left), and lower binding affinity (Figure 4C, second from bottom left) compared to OM. Additionally, it also appears that that while drug mutations need not necessarily occur at the hydrophobic sites (KD values, *P* > 0.05), they tend to lie buried indicated by higher RD values, and consequently lower surface area (ASA and RSA) compared to OM (Figures 4A,B).

### Distinct Stability Profile for Drug Mutations and Lineage 1

**FIGURE 7:**
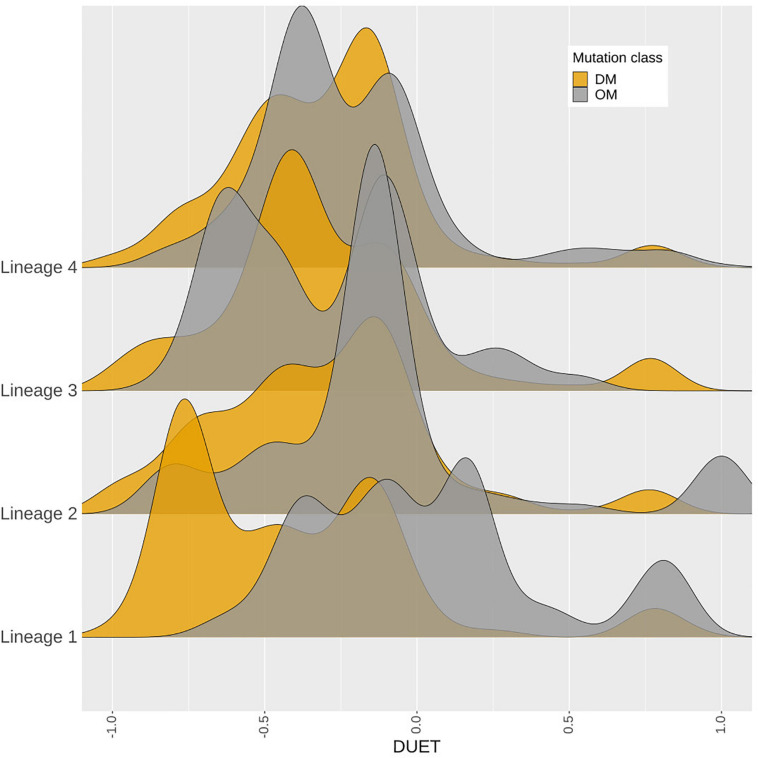
Density distribution of *M. tuberculosis* lineages. A total of 4,433 samples belonging to Lineages 1–4, containing 419‘pncA mutations were considered. The horizontal axis shows the DUET stability values (−1, most destabilising) to blue (+1, most stabilising). while the vertical axis shows the density distribution of *M. tuberculosis* lineages coloured by mutation class as either DM (associated with pyrazinamide resistance in orange) or OM (not associated with pyrazinamide drug resistance which appear in grey). DM mutations comprise of a total of 3,565 samples contributing to 226 mutations, while 868 samples contributing to 193 mutations formed part of the OM mutation class. The figure is generated using R statistical software (version 4.0.2). Abbreviations used: nsSNPs: non-synonymous Single Nucleotide Polymorphisms, pncA: pyrazinamidase.

A total of 419 nsSNPs are lineage specific (L1: 74; L2: 277; L3: 104; L4: 311). The greatest diversity of nsSNPs was observed in L3 (54.7%), followed by L1 (51.4%) and Lineage 2 (14.7%) with L4 showing the lowest diversity (14.1%) despite containing the highest number of samples (Supplementary Figure 6). Statistical analysis of the DUET ΔΔG distributions revealed significant differences between all lineages except between L3 and L4. Lineage differences for DUET ΔΔG were most prominent between L2 and L4 (*P* < 0.0001), followed by L1 and L4 (*P* < 0.001) (Supplementary Table 2A). Within each lineage, mutational distributions were significantly different between DM and OM mutation classes (*P* < 0.0001) except L3 (Supplementary Table 2B). Interestingly, a distinct stability profile was observed for DM mutations within L1. Mutations associated with drug resistance showed a marked peak around the extreme end (−0.75 DUET ΔΔG) of the destabilising spectrum (Figure 7) within L1.

## Discussion

Genetic mutations including nsSNPs present within drug-targets and their activating genes are the main drivers of resistance development in TB (Schön et al., 2017). The motivation for investigating the missense mutations within the protein coding region only of the *pncA* gene was to enable understanding of the phenotypic mutational effects in relation to PZA resistance development. While the exact molecular mechanisms of PZA resistance are yet to be fully elucidated, the binding pocket of PZA and its key interactions are well known and characterised (Petrella et al., 2011; Ali et al., 2020; Sheik Amamuddy et al., 2020; Khan et al., 2021). This knowledge was used to guide the molecular docking of PZA to generate the pncA-PZA complex in the absence of an experimentally solved structure of the bound complex in Mtb. While docking generates a variety of ligand conformations (poses), choosing the “best” pose is based on considerations around key molecular interactions formed by the ligand, interaction energy of the docked complex and subject expertise. Using these guides, docking pose 1 was chosen due to its molecular interactions with known key residues and close alignment with previously published studies (Karmakar et al., 2018; Ali et al., 2020; Khan et al., 2021). In addition, we analysed the top two docking poses using the mCSM pipeline (Supplementary Figure 3). The resulting mutational effects on pncA stability and ligand affinity did not differ between poses indicating the small differences in pose did not affect downstream analysis. It also suggests that due to the small size of the PZA molecule, the orientation of the aromatic ring within the cavity may have more flexibility in its orientation and interaction with the neighbouring residues, but without drastically impacting the molecular interactions for global protomer stability and ligand binding affinity.

The molecular motion of pncA assessed by NMA was visualised to understand the mutational effects with regard to flexibility (Supplementary Figure 1). Sites displaying high mutational frequency or association with drug resistance mutations were not located in regions with high flexibility, with large molecular motions mainly restricted to the loop region 60–66. This suggests the molecular motion in pncA does not interfere with PZA binding as active site residues were not associated with high fluctuations.

Normal mode analysis shows large scale molecular motions. Molecular dynamics (MD) studies offer insights into the finer grained atomic motions and are an excellent way to investigate molecular mechanisms. However, these studies are computationally intensive and are difficult to scale for studying hundreds of mutations. A recent MD study on a subset of mutations found within our dataset analysed seven pncA nsSNPs (F94L, F94S, K96N, K96R, G97C, G97D, and G97S) showed that these destabilising mutations altered the binding pocket, allowing increased PZA flexibility (Khan et al., 2021). All seven mutations were associated with PZA resistance and also showed destabilising effects in our study. A similar study of destabilising mutations R123P, T76P, H7R associated with PZA resistance showed that the mechanism of resistance could be through increasing the flexibility of the region they are located in, thereby changing the binding pocket volume (Ali et al., 2020). Another MD study of mutations P54L and H57P showed that they decrease overall stability along with reduced ligand affinity leading to PZA resistance (Mehmood et al., 2019). All of these observations are concordant with our analysis.

Destabilising effects of nsSNPs are thought to be the main reason for impeding protein function through directly effecting protomer stability or ligand affinity. However, large stabilising effects can have an equally deleterious impact on protein function through rigidification, impeding flexibility and dynamic molecular motions. This has been implicated more generally within a disease context (Gerasimavicius et al., 2020) and more specifically in PZA resistance (Rajendran and Sethumadhavan, 2014). It offers an explanation for the observance of the stabilising mutation site 103. Drug associated mutations at this site (Y103C, Y103H, and Y103S) could result from the rigidification of the binding pocket leading to reduced binding affinity measured as destabilising PZA affinity.

Mutations within *pnca* are scattered along the entire gene length observed in studies (Stoffels et al., 2012; Miotto et al., 2014; Whitfield et al., 2015). While two other genes, *rpsA* and *panD* have also been linked to PZA resistance, a clear link between *rpsA* and PZA resistance is lacking (Shi et al., 2011; Alexander et al., 2012; Simons et al., 2013; Tan et al., 2014) although there is increasing evidence to support *panDs* association with PZA resistance (Pandey et al., 2016; Werngren et al., 2017; Gopal et al., 2020). In our analysis, there were only a few samples with *rpsA* and *panD* mutations, therefore limiting attempts at assessing their synergistic relationship with PZA resistance. Mutations within the *pncA* gene and its promoter remain the most common route to PZA resistance (Dookie et al., 2018) (Khan et al., 2019). Nearly 70% of the MDR isolates and 13% XDR isolates had nsSNPs in the *pncA* coding region. The burden of pncA mutations in the MDR and XDR isolates was lower in our analysis compared to 88.0% and ∼20% observed by Pang et al. (2017). In another study, 70% of the MDR isolates, and significantly higher i.e., 96% of XDR isolates harboured pncA mutations including nsSNPs (Allana et al., 2017). An alternative route to resistance for pncA as a non-essential gene encoding an enzyme that transforms a prodrug to drug would be by INDELs or mutations leading to premature stop codons resulting in the protein being degraded on translation. A recent report analysing the *pncAc.85_86insG* frameshift mutation using structural and biophysical analysis showed the mutation resulted in a truncated and incomplete protein lacking the active site pocket (Karmakar et al., 2018). Despite this obvious route to resistance, only 1% samples in our dataset showed INDELs and stop codons, compared to 13% of samples that showed missense point mutations in pncA. This is consistent with the knowledge that nsSNPs in pncA remain the major route to resistance for PZA (Khan et al., 2019).

Destabilising effects are considered detrimental to the downstream protein function (via disruption of drug affinity, nucleic acid affinity or overall complex stability) and are thus given higher consideration in classifying mutations (Wylie and Shakhnovich, 2011). In our analysis, around 85% of mutations were destabilising for overall protein stability as well as complex affinity. It is thought that the resistant phenotype is imparted either through affecting protein folding, instability of the PZase protein, prevention of coenzyme complex (Gopal et al., 2016) or loss of virulence factor synthesis (Gopal et al., 2016). Further, this is thought to come without a high bacterial fitness cost since pncA is primarily an activator of the PZA drug. This is similar to a recent observation reported in the *katG* gene (target for the anti-TB pro-drug, isoniazid) with a high proportion of destabilising mutations (Portelli et al., 2018). Also, a higher proportion 60% (*n* = 253) of SNP mutations showed electrostatic changes compared to ∼35% reported by Portelli et al. (2018). This likely due to the larger sample size of our dataset.

All active site residues appear to be under drug selection pressures due to multiple mutations (>2) associated with these with the exception of I133, considered to be an emerging or budding-resistance hotspot. In our analyses, there were 22 such sites while 83 sites within *pncA a*ssociated with > 2 nsSNPs linked to PZA drug resistance (categorised as DM). However mutations were not restricted to the active site, with less than 50% resistant variants lying within 10 Å of the active site of PZA, indicating the possible role of distal residues in resistance development (Portelli et al., 2018). Mutations associated with drug resistance tend to have lower stability, lie buried within the structure with lesser surface area as shown by Karmakar et al. (2020).

Our study compares results from two different computational stability predictors: mCSM and FoldX (Schymkowitz et al., 2005). Unsurprisingly, most mutations were found to have a destabilising effect (Supplementary Figure 11). FoldX reported ∼85% (vs. ∼80% estimated by DUET) nsSNPs with destabilising effect. The range for absolute ΔΔG values was greater for FoldX (median: 2.0; range: −5.2, 27.46) compared to DUET (median: −0.1; range: −3.9, 1.2). There was however, 77% agreement between FoldX and DUET outcomes (data not shown). Interestingly, drug associated mutations displayed higher FoldX ΔΔG predictions compared to mCSM-DUET ΔΔG predictions. A possible explanation for this is the differences in the underlying parameters the different methods use. FoldX constructs mutant structures by mutating the target residue and searching for the optimal conformation by iteratively altering the position of the neighbouring side chains. The stability of the mutant structure is estimated using an empirical force field made of several energy terms. This compares to DUET where estimates of the structural effects are based on differences between the wild-type environment and pharmacophore atomic changes resulting from the mutation, without the need to generate mutant structures. With this in mind, it appears that the DM mutations have larger local perturbations in the mutated region considered by FoldX, resulting in higher ΔΔG predictions compared to the lesser effects of surface area considered by DUET. Drug resistance mutations displaying smaller surface area compared to their susceptible counterparts were also observed in recent studies investigating nsSNPs in Mtb genes (Portelli et al., 2018; Karmakar et al., 2020) indicating the role of compensatory mutations, alleviating any fitness penalty in the development of the drug resistance phenotype. The extent of the contribution of surface area in these methods is reflected in the observation of moderate correlations between DUET and structural features, and the weaker associations between FoldX and structural features (Supplementary Figure 9A). Structural associations for ligand affinity were also observed to be weak (Supplementary Figure 9B) most likely due to the role of factors involved in short-range interactions (like Van der Waal’s forces) not considered in our analysis. A similar view emerged in the recent study by Karmakar et al. (2020) where no significant differences were observed for PZA binding affinity.

It has been suggested that frequently occurring mutations may not confer extreme changes in biophysical stability measures, with mild stability effects offering local fitness advantages (Portelli et al., 2018). Our data presented us with the opportunity to test this theory empirically by assessing relationships of stability with GWAS measures of MAF, OR, and *P*-values. At a glance, it appears that mutations with high OR tend be less extreme in their impact on protein stability and ligand affinity (Supplementary Figure 10). However, we did not find any significant association with high frequency mutations and extreme changes in stability or affinity parameters (Figure 6). One possible explanation is that the fitness landscape is gene and function specific, optimised differently for genes directly coding for drug targets and for non-essential genes like *pncA*. Another major consideration is that resistance is often acquired through a stepwise ordinal accumulation of mutations (Woodford and Ellington, 2007; Ismail et al., 2019). The genetic background can dramatically influence fitness effects associated with mutations (Wong, 2017). Consequently, the mutational impact differs when occurring against a sequence background of extant resistant mutations, a phenomenon known as epistasis (Wong, 2017). Since resistance development is a balanced interplay between fitness effects and cost of resistance, epistasis warrants due consideration in efforts to understand and limit the evolution of multi-drug resistance.

The use of mCSM suite of tools has the advantage of studying global (protein stability) as well as local effects (ligand affinity, protein-protein interaction, and protein nucleic-acid interaction). Additionally, it also provides the methodological consistency for comparing molecular effects and benefits application of machine learning methods (ML) to explore greater mechanistic details. While computationally intensive, ML methods would benefit from using tools such as DynaMut (Rodrigues et al., 2018) which account for protein molecular motions when estimating mutational effect on protein stability. Additionally methods which consider anti-symmetric properties of mutational impact i.e., ΔΔG (A → B) = −ΔΔG (B → A) like DeepDDG (Cao et al., 2019) and INPS-MD (Savojardo et al., 2016) have the potential to build robust predictive models and improve the “learning” capability of ML methods in the context of machine learning.

Mtb lineages have been associated with virulence, disease transmission, drug resistance, and clinical outcome (Ford et al., 2013; Reiling et al., 2013; Novais et al., 2017; Correa-Macedo et al., 2019; Oppong et al., 2019; McHenry et al., 2020). Lineage specific differences between lineages 2 and 4 have recently been noted in the development of TB drug resistance, especially related to MDR and XDR strains (Oppong et al., 2019). Our study highlighted the most significant differences between L2 and L4 with respect to protomer stability demonstrating the biophysical phenotypic manifestation of these underlying genotypic changes. The observance of a distinct peak for destabilising mutations related to drug resistance within L1 suggests that the extreme mutational consequences of such mutations in the “ancient” lineage 1 may be rapidly giving way to other “modern” *M. tuberculosis* lineages linked to MDR and XDR-TB and virulence.

Our study is based on a well-characterised clinical dataset sourced globally from over 35 K clinical isolates, and leverages the availability of robust metadata (lineage, geography, DST, etc.) for each isolate. We show that the framework used in our work allows us to investigate the interrelationships between genomic features from GWAS analysis and the biophysical measures of nsSNPs, helping to contextualise the underlying bacterial fitness and mutational landscape. The need to consider multiple stability predictors with different underlying principles to validate these associations has also been highlighted. Lineage associations of drug resistance, and their biophysical consequences, require further investigation and the functional characteristics of mutations should be validated in future experiments. We hope such a framework can be used to understand and inform therapeutic and stewardship efforts.

## Data Availability Statement

All pre-generated TB-profiler results were downloaded for all isolates from tbdr.lshtm.ac.uk/sra.

## Author Contributions

TT was responsible for the molecular docking, integrating genomics and structural data, data analysis, and writing the initial draft. JP made available and generated the genomics data results. CE provided the FoldX pipeline. TC and NF provided the overall supervision and contributed to revising and refining the manuscript. All authors contributed to the manuscript and approved the submitted version.

## Conflict of Interest

The authors declare that the research was conducted in the absence of any commercial or financial relationships that could be construed as a potential conflict of interest.
