# Supplementary material for: Structural and Genomic Insights Into Pyrazinamide Resistance in Mycobacterium tuberculosis Underlie Differences Between Ancient and Modern Lineages
Source: Front Mol Biosci. 2021 Jul 23;8:619403. doi: 10.3389/fmolb.2021.619403 (PMC8372558; doi:10.3389/fmolb.2021.619403)
Supplement: Supplementary file 3 [file Data_Sheet_1.docx]

**Structural and genomic insights into pyrazinamide resistance in *Mycobacterium tuberculosis* underlie differences between ancient and modern lineages**

Tanushree Tunstall^1^, Jody Phelan^1^, Charlotte Eccleston^1^, Taane G. Clark^1,2^, Nicholas Furnham^1^

1. Department of Infection Biology, London School of Hygiene and Tropical Medicine, Keppel Street, London, WC1E 7HT, UK.

2. Department of Infectious Disease Epidemiology, London School of Hygiene and Tropical Medicine, Keppel Street, London, WC1E 7HT, UK.

**Supplementary Figures 1-11**


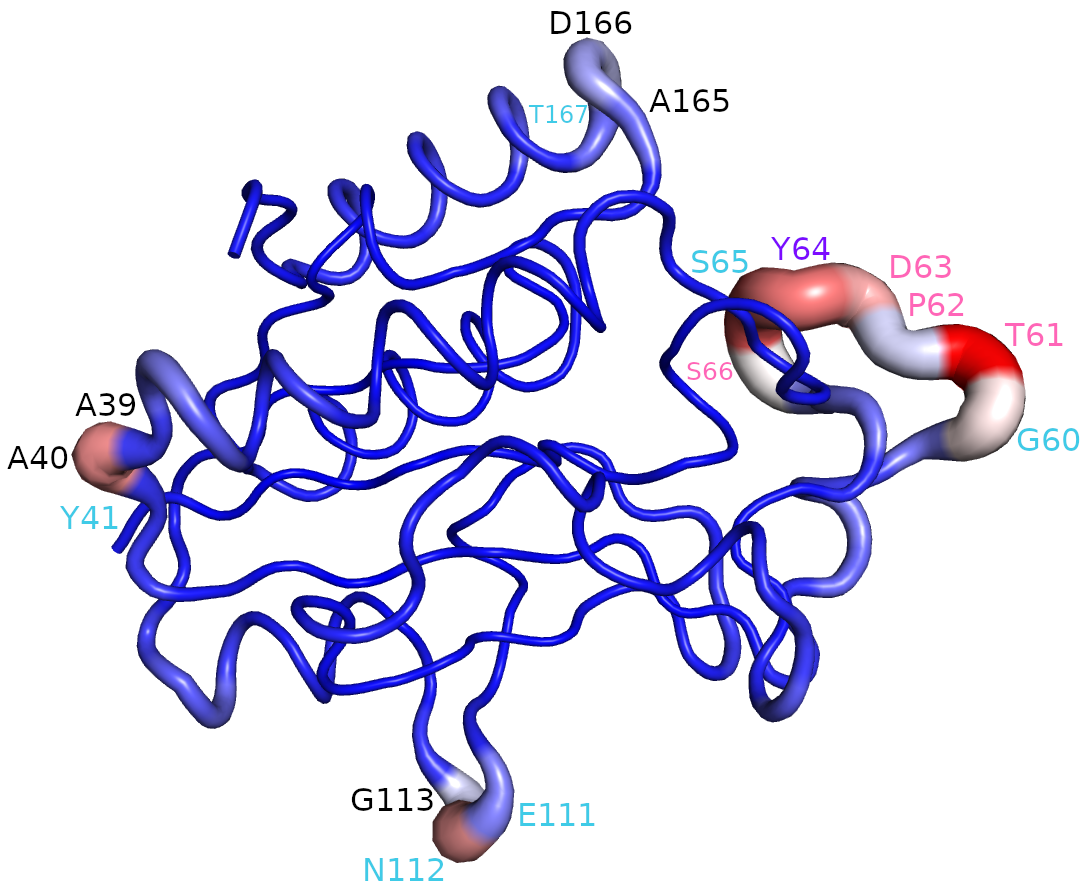


### Supplementary Figure 1. Protein fluctuation analysis of pncA structure (3PL1) based on Normal Mode Analysis (NMA).

Sites associated with fluctuation as depicted by NMA. The magnitude of the fluctuation is represented by thin to thick tube coloured blue (low), white (moderate) and red (high). The corresponding wild-type residues (using the standard one-letter code) at these sites are labelled and coloured according to the mutational effects of one or more nsSNPs at these sites: Drug resistant mutations (DM) are coloured purple, Other mutations (OM) appear in blue, while sites linked to mutations belonging to either category are coloured in pink. Sites associated with no nsSNPs in our study are depicted in black. The NMA analysis and figure is generated from the DynaMut web server. Abbreviations used: pncA: pyrazinamidase.


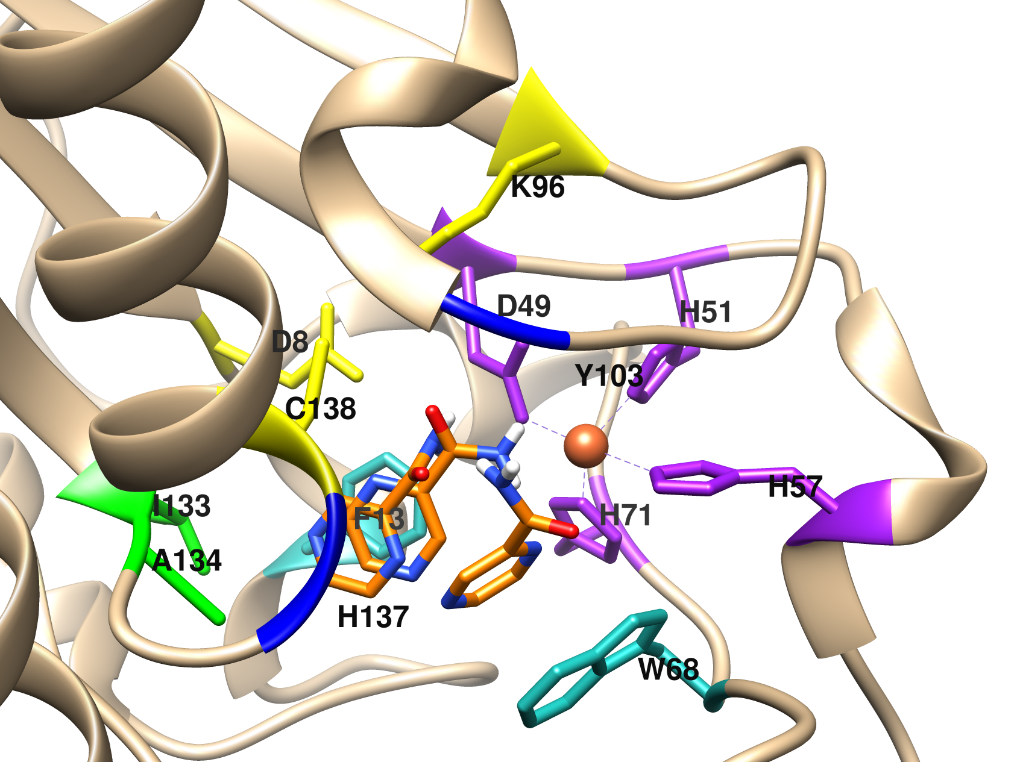


- - 1. **B**
    2. **A**

###
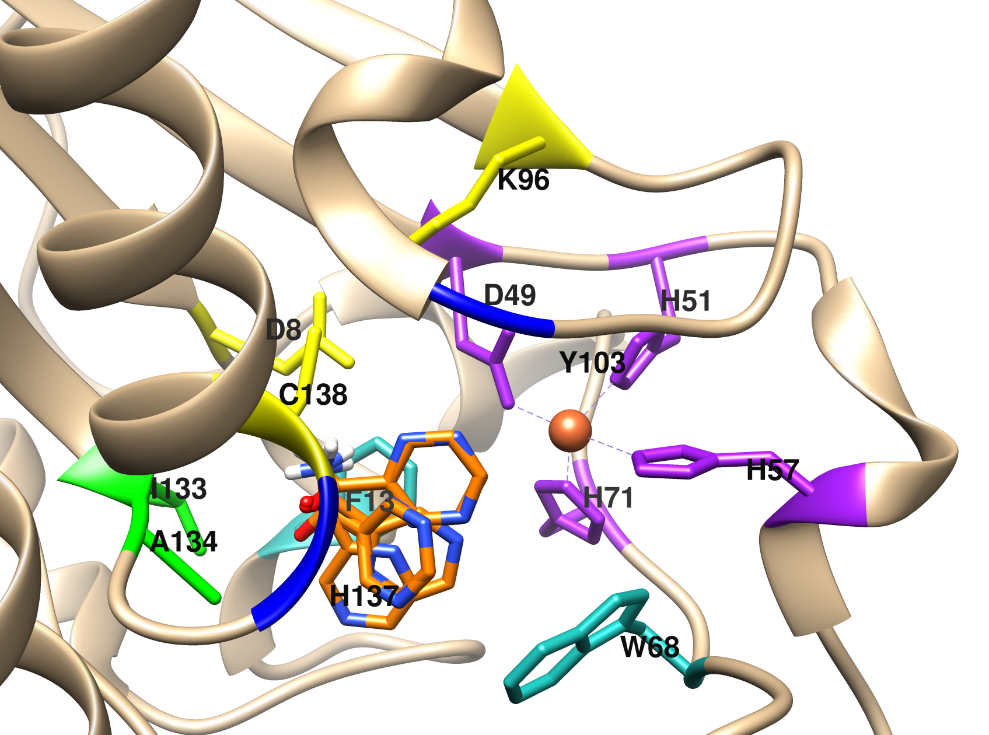


### Supplementary Figure 2. Docking of Pyrazinamide (PZA) within pncA

Configuration of nine PZA poses returned by Autodock Vina located within the binding cavity, exploiting confirmations around the one rotatable bond in PZA. (**A)** Poses 1, and 3-6 with orientation of PZA ring towards the ring of tryptophan (W68), while **(B)** Poses 2, 8 and 9 showing the orientation of the PZA ring away from tryptophan. Residues marked in green participate in hydrogen bonding, residues in yellow form the catalytic triad, residues in teal (and blue) are involved in substrate binding, while residues in purple are involved in the iron centre. The figure is rendered using Chimera (version 1.14).


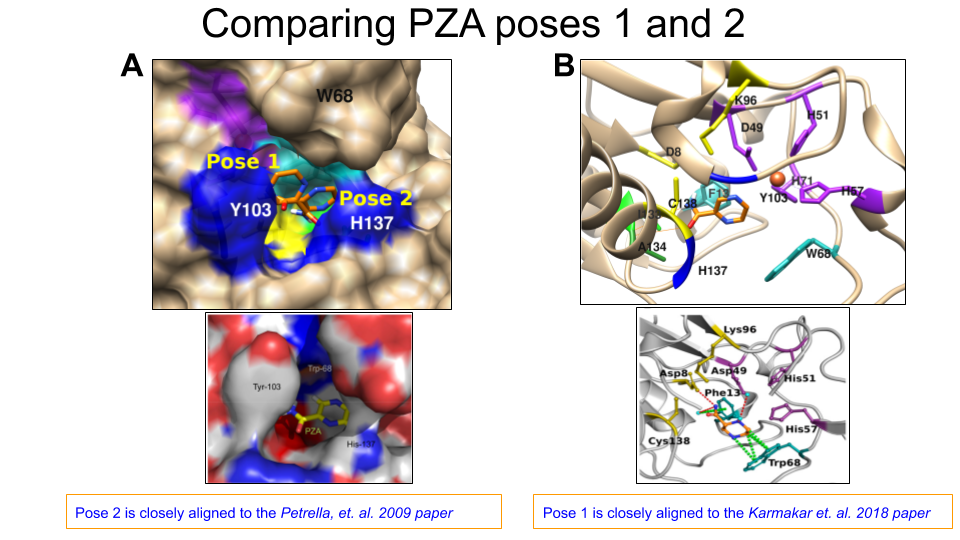


### Supplementary Figure 3. Comparing PZA Poses 1 and 2 in relation to docking

**(A)** Comparison of poses 1 and 2 returned from Autodock Vina highlighting the differing orientation of the ring between the two poses.**(B)** Pose 1 resembles closely to the docking performed in the recent case report published (Karmakar et al., 2018), while pose 2 is closely aligned with the proposed binding cavity by the authors of the pncA crystal structure (Petrella et al., 2011).

###

- - 1. **pncA complex with pose 1**
    2. **A**
    3. **pncA complex with pose 2**
    4. **B**

###
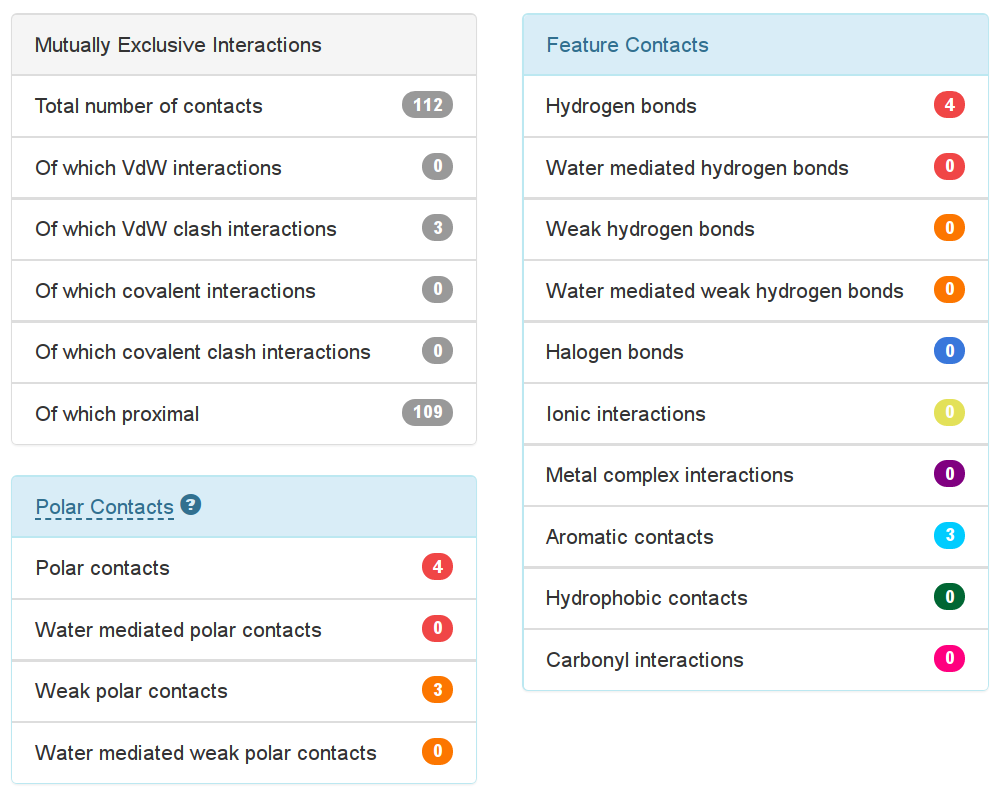

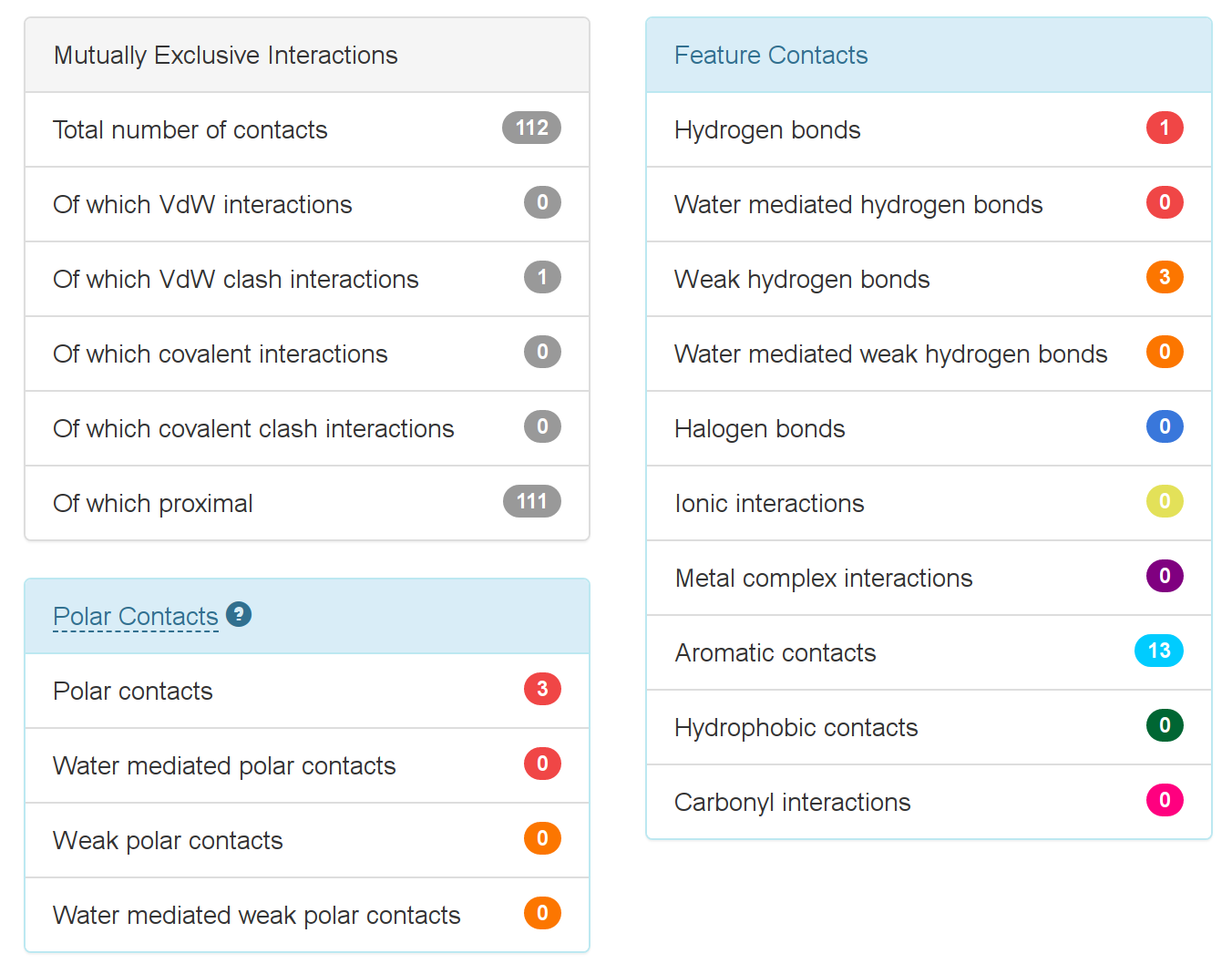


### Supplementary Figure 4. Molecular interactions for PZA Poses 1 and 2

Arpreggio analyses showing molecular interactions between PZA pose1 (**A)** and **(B)** pose 2 reporting differences between hydrogen bonds, aromatic contacts, polar contacts and Van der Waals interactions. Screenshot from Arpgeggio web server (Jubb et al., 2017).

###
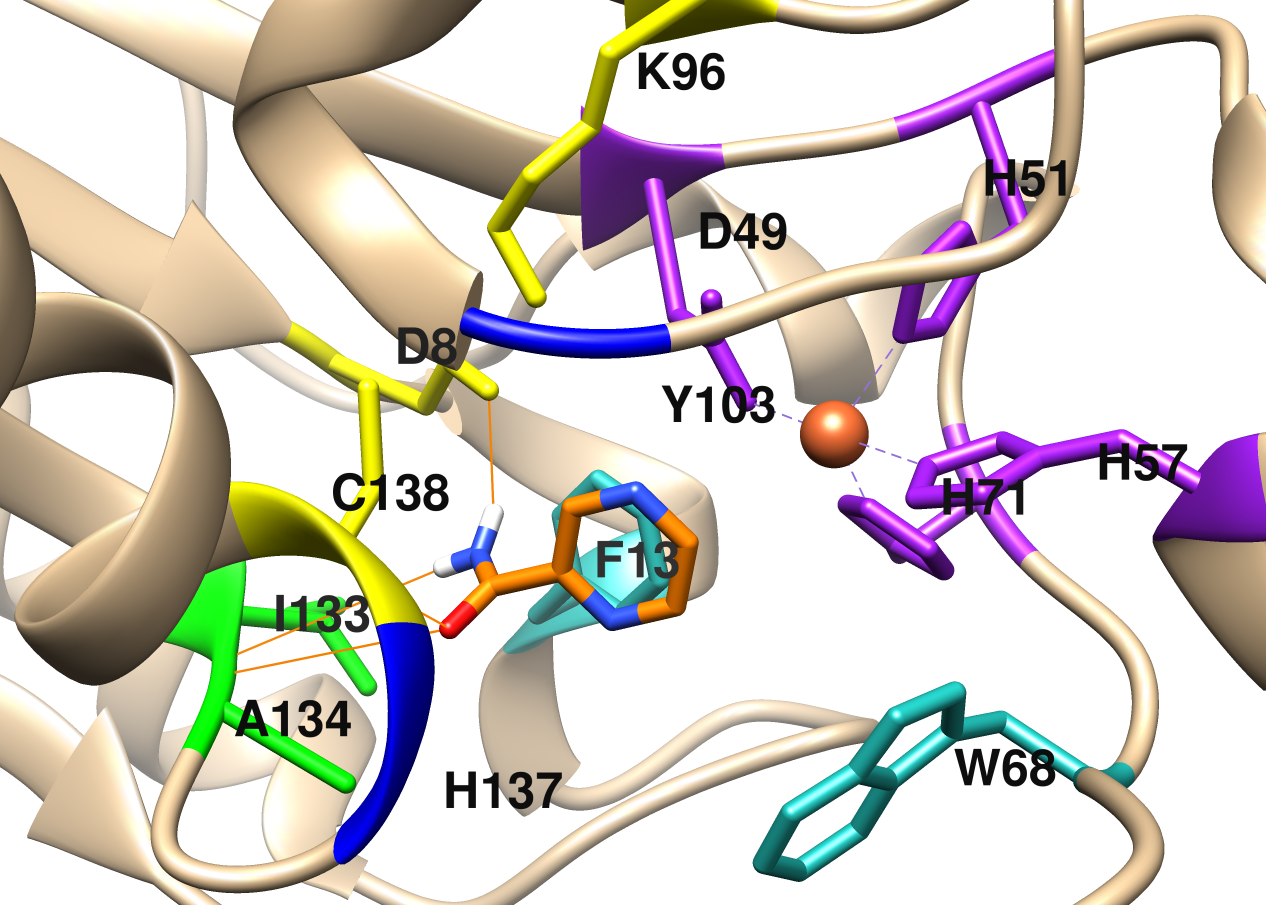
 Supplementary Figure 5. Molecular docking of PZA with pncA

Protein-ligand complex formed by pncA with pose 1 of PZA after docking. Residues marked in yellow form the catalytic triad, residues in teal and blue are involved in substrate binding, while residues in purple are involved in the iron centre. Residues marked in green participate in hydrogen bonding, with hydrogen bonds between PZA and D8, I133, A134 and C138 are shown in orange. The figure is rendered using UCSF Chimera (version 1.14).

###
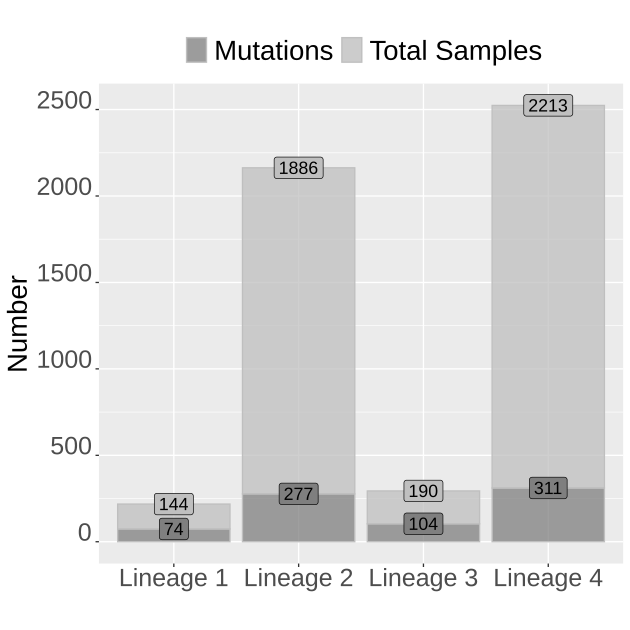
Supplementary Figure 6. Barplot of total samples and mutations within Mtb Lineages

The total number of samples along with the number of mutations associated with PZA resistance within the 4 Mtb Lineages. The dark grey bars show the number mutations, while the light grey bar show the total number of samples within each lineage. Lineage 1 has 74 mutations out of 144 samples, Lineage 2 has 277 mutations out of 1886 samples, while lineages 3 and 4 have 104 and 311 mutations out of 190 and 2213 number of samples respectively. The figure is generated using R statistical software (version 4.0.2).

###
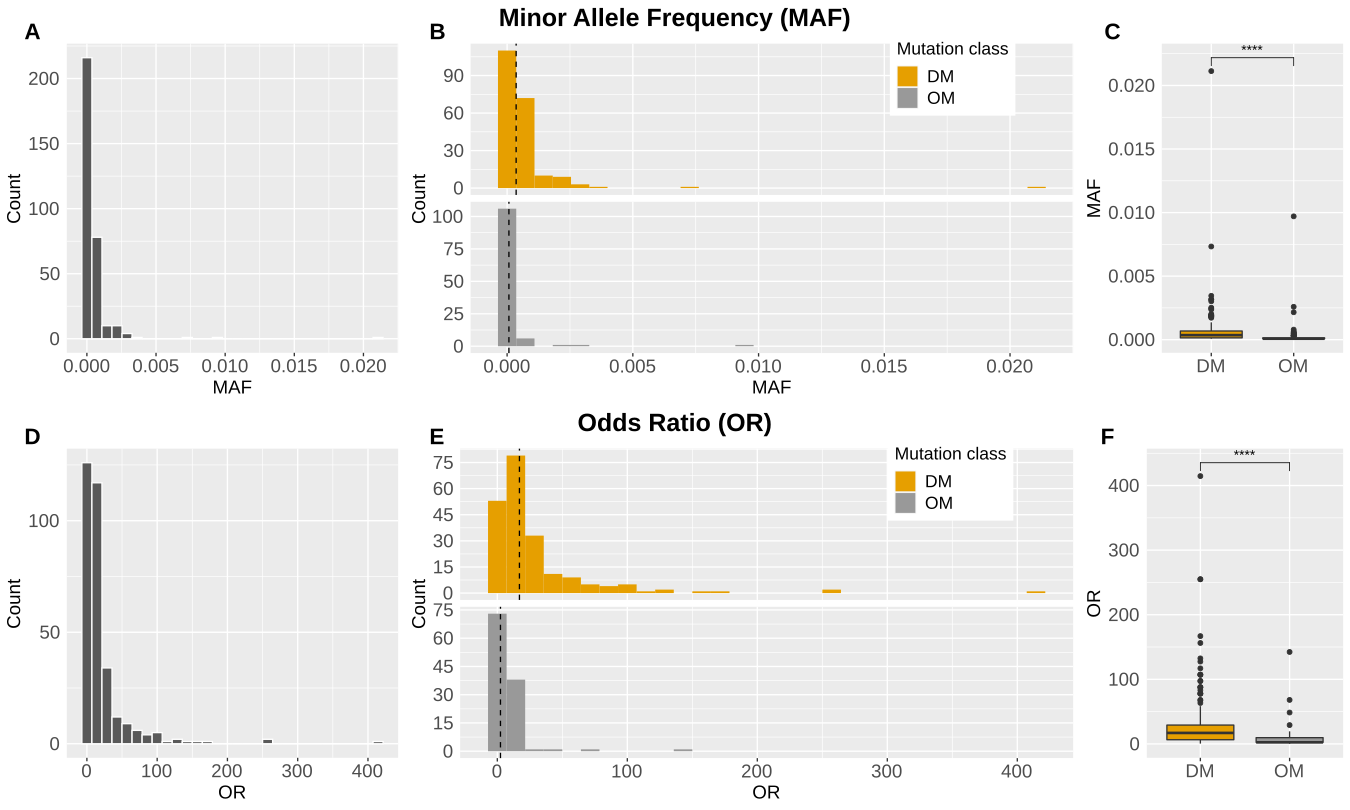
**Supplementary Figure 7. Frequency distribution of Minor Allele Frequency (MAF) and Odds Ratio (OR) for pncA SNP mutations.**

MAF and OR were calculated for a total of 322 nsSNPs. The top panel relates to Minor Allele frequency where (A) Histogram of MAF, (B) Histogram of MAF according to mutation class as either DM (associated with pyrazinamide resistance coloured in orange) or OM (not associated with pyrazinamide drug resistance coloured in grey). Dashed lines indicate median. (C) Box plot comparing MAF between DM and OM mutations. The bottom panel relates to Odds Ratio where (D) Histogram of OR, (E) Histogram of OR according to mutation class: ‘DM’ in orange and ‘OM’ in grey. Dashed lines represent median, (F) Boxplot comparing OR between DM and OM mutations. Wilcoxon rank-sum (unpaired) test was used to compare DM and OM mutations, and significance indicated as ****P<0.0001. The figure is generated and statistical analysis performed using R statistical software (version 4.0.2).

###
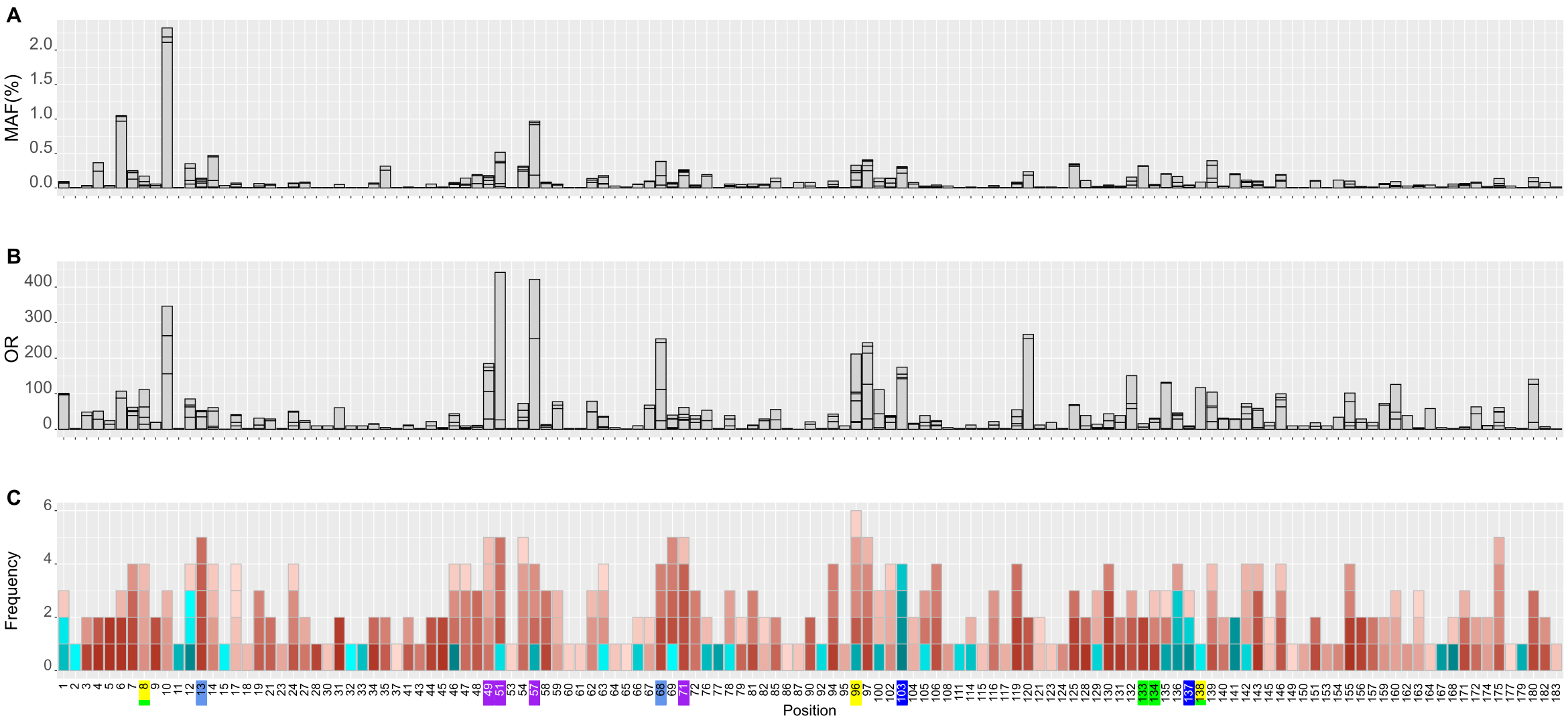
Supplementary Figure 8. Minor Allele Frequency (AF), Odds Ratio (OR) and DUET effects of nsSNPs within pncA

Barplot showing 322 mutations associated with MAF, OR and SNP diversity by position highlighting the prominent positions in terms of MAF. OR and frequency of nsSNPs within pncA. The horizontal axis shows the mutational positions within pncA and are coloured as green (residues involved in hydrogen bonding with PZA) yellow (catalytic triad), blue and teal (substrate binding), purple (iron centre).The vertical axis shows **(A)** cumulative MAF associated with one or more mutations at that position, **(B)** cumulative OR associated with one or more mutations at the given position and **(C)** the frequency of nsSNPs at mutational position within pncA. The red and the blue bars denote destabilising (n=279) and stabilising (n=43) mutations for a total of 322 mutations according to DUET. Destabilising mutations are depicted in red and stabilising mutations in blue, where colour intensity reflects the extent of effect, ranging from -1 (most destabilising) to +1 (most stabilising). The figure is generated using R statistical software (version 4.0.2).

###
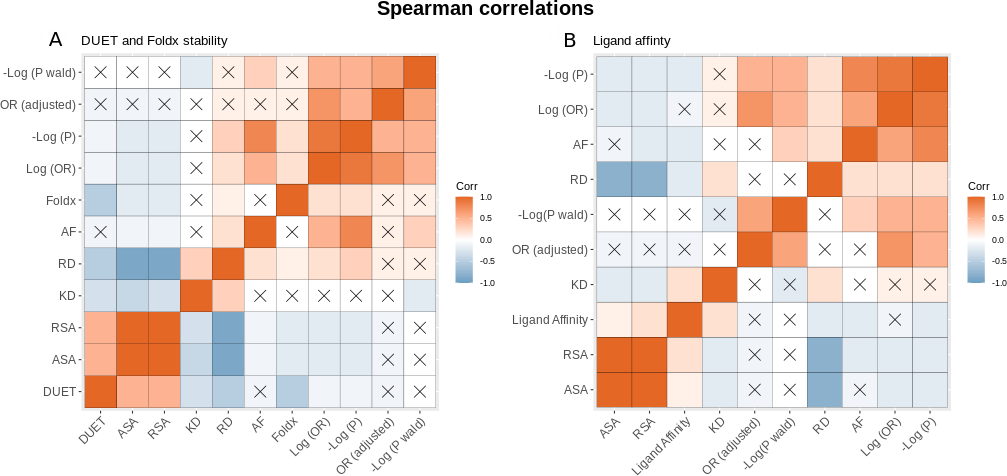
Supplementary Figure 9. Correlation of protomer stability and ligand affinity effects with GWAS and structural parameters.

Pairwise Spearman correlations between Foldx stability, DUET stability, Allele Frequency (AF), negative log P-value (-Log(P)), Log Odds Ratio (OR), adjusted Log OR (accounting for sample relatedness in GWAS analysis), negative log P-value from Wald test corresponding to the adjusted OR, along with structural parameters of accessible (ASA) and relative (RSA) surface area, KD (hydrophobicity values based on the Kyte and Doolittle scale) and RD (Residue Depth). The parameters are ordered using hierarchical clustering. Squares marked with an ‘X’ indicate statistical insignificance (P*>*0.05). Part (A) shows correlations with DUET and Foldx stability values, for a total of 424 nsSNPs, while (B) shows correlations with Ligand affinity for a 201 nsSNPs. The figure is generated using R statistical software (version 4.0.2).

- - 1. **
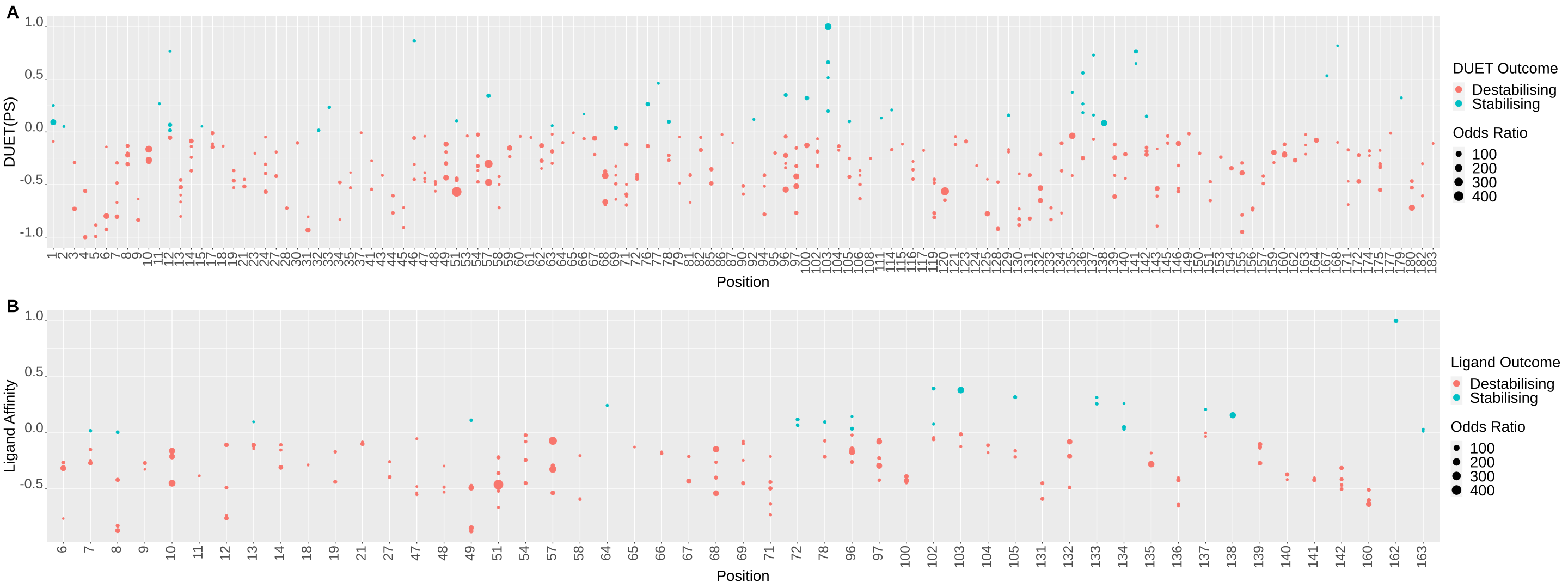
**

### Supplementary Figure 10. Comparing stability effects of nsSNPs with GWAS measures of Odds Ratio (OR)

Bubble plot displaying the relationship between OR with **(A)** DUET Protein stability and **(B)** Ligand affinity corresponding to and 322 and 160 mutations respectively. The horizontal axis shows the mutational positions within pncA and the vertical axis shows protein stability effects ranging from -1 (most destabilising) to +1 (most stabilising). Each dot represents a unique mutation at that position, with the colour corresponding to destabilising (red) and stabilising (blue) mutations, while the size of the dot is proportional to the OR of that mutation. The figure is generated using R statistical software (version 4.0.2).

### Supplementary Figure 11. Barplot of mutations with protein stability effect according to FoldX.


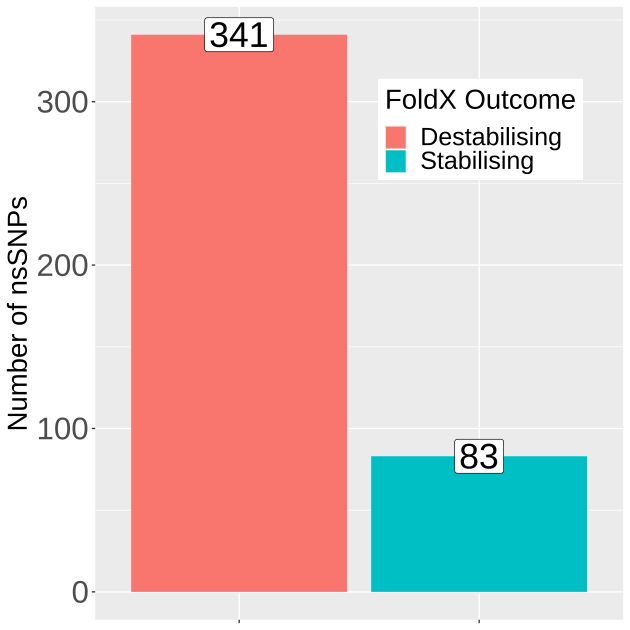
Number of mutations (nsSNPs) categorised as destabilising (n=341) and stabilising (n=83). The figure is generated using R statistical software (version 4.0.2).
